# Unravelling Mixed Organic‐Halide Perovskite Degradation Under Extrinsic Factors

**DOI:** 10.1002/smll.202509525

**Published:** 2025-12-08

**Authors:** Manuel Salado, Timur V. Tropin, Abdessamad El Adel, Lisa Sarah Fruhner, Julia Sánchez‐Bodón, Jose L. Vilas‐Vilela, Anton P. Le Brun, Thomas Saerbeck, Ivan Infante, Viktor Petrenko, Jose M. Porro

**Affiliations:** ^1^ Basque Center for Materials Applications and Nanostructures BCMaterials Bld. Martina Casiano, UPV/EHU Science Park, Barrio Sarriena, s/n Leioa 48940 Spain; ^2^ IKERBASQUE Basque Foundation for Science Bilbao 48013 Spain; ^3^ Jülich Centre for Neutron Science (JCNS‐1) and Institute for Complex Systems (ICS‐1) Forschungszentrum Jülich GmbH Leo‐Brandt‐Straße 52425 Jülich Germany; ^4^ Macromolecular Chemistry Group (LABQUIMAC) Department of Physical Chemistry Faculty of Science and Technology University of the Basque Country (UPV/EHU) Barrio Sarriena s/n Leioa E‐48940 Spain; ^5^ Australian Centre for Neutron Scattering Australian Nuclear Science and Technology Organisation Lucas Heights Sydney NSW 2234 Australia; ^6^ Institut Laue‐Langevin 71 Avenue des Martyrs Grenoble Cedex 9 38042 France

**Keywords:** ionic diffusion, neutron reflectometry, perovskite, solar cells, stability

## Abstract

Over time, halide perovskite materials used in solar cell applications, can experience several degradation mechanisms, including moisture ingress, thermal stress, light‐induced degradation, and ion migration, all of which lead to reduced performance and stability in devices. Among these, moisture ingress is particularly critical for the stability of perovskite solar cells. The perovskite structure is highly sensitive to water molecules, which can trigger chemical reactions and phase transitions. While significant progress has been made in mitigating perovskite degradation—through strategies such as interface engineering, encapsulation techniques, and compositional optimization—further research is necessary to develop perovskite solar cells with the long‐term stability required for commercial use. Advanced characterization techniques like neutron reflectometry (NR) offer valuable insights into degradation mechanisms by using isotope substitution to track specific components within the material. For that, this work presents first, how affect the humidity and temperature in the full device, second, their characterization to unravel the degradation mechanism with NR and finally, corroborates the results with simulation techniques. NR results suggest enhanced stability of hybrid perovskite films deposited on TiO_2_ layers, and indicate the formation of interfacial layers at the base of the film, likely composed of FAI, PbI_2_, and MABr. The obtained experimental results are supported by molecular dynamics simulations modelling.

## Graphical abstract

Advanced characterization methods like neutron reflectometry (NR) provide insights into degradation by using isotope substitution to track components. NR indicates improved stability of hybrid perovskite films on TiO_2_ and reveals interfacial layers at the film base, likely containing FAI, PbI_2_, and MABr. Molecular dynamics simulations support these experimental findings under various environmental conditions to better understand material performance and stability.

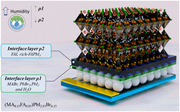

## 1 Introduction

The development of perovskite solar cells (PSCs) has become a pivotal area of research in the field of photovoltaics. After a decade of intensive study, it is well‐established that PSC technology offers substantial potential for enhancing solar energy production.^[^ ^1^ ^]^ Its exceptional optical properties, straightforward fabrication process, and low‐cost materials make PSCs a highly competitive alternative to traditional silicon‐based solar cells.^[^ ^2^ ^]^ Additionally, the tunability of perovskite materials allows for high power conversion efficiency, and when used in tandem with silicon solar cells, it produces synergistic effects that further boost performance. Despite these impressive attributes, the long‐term stability of PSCs remains a significant barrier to their large‐scale industrial application. Four key external factors—humidity, temperature, oxygen, and light—alone or in combination, initiate degradation reactions that lead to the loss of electro‐optical properties.^[^ ^3^ ^]^ For example, thermal stress during both fabrication and operation can cause structural defects, grain boundary migration, and compositional changes.^[^ ^4^ ^]^ Similarly, light‐induced degradation (or light soaking)^[^ ^5^ ^]^ results from the generation of charge carriers that interact with defects and impurities, forming trap states that reduce charge extraction efficiency. To address these challenges, considerable research efforts have focused on understanding the degradation mechanisms within the perovskite structure and how external factors accelerate these processes.^[^ ^6^, ^7^, ^8^, ^9^, ^10^ ^]^ Wang et al.^[^ ^11^ ^]^ conducted a seminal study outlining the potential degradation reactions occurring during perovskite decomposition under various environmental conditions. However, the wide variety of perovskite compositions and device configurations has made it difficult to reach a consensus on the exact degradation pathways. For instance, mixed perovskite compositions (e.g., Cs_0.05_(MA_0.15_FA_0.85_)Pb(I_2.7_Br_0.3_)) have demonstrated greater stability compared to single‐cation perovskites (e.g., MAPbI_3_, FAPbI_3_, CsPbI_3_), where MA represents methylammonium, and FA represents formamidinium.^[^ ^12^, ^13^ ^]^ Nevertheless, some controversy persists, particularly when comparing planar PSC configurations (without an electron transport layer) to mesoporous designs incorporating materials like SnO_2_ or TiO_2_.^[^ ^14^, ^15^, ^16^, ^17^, ^18^, ^19^, ^20^ ^]^ Some studies suggest that oxide‐based layers in mesoporous configurations can exhibit photocatalytic activity under natural light and ultraviolet (UV) exposure, potentially contributing to device degradation.^[^ ^21^ ^]^

In recent years, various techniques have been employed to investigate and elucidate the intrinsic degradation mechanisms of perovskite devices. These methods include Photoluminescence Spectroscopy (PL), Impedance Spectroscopy (IS), Nuclear Magnetic Resonance (NMR), and several X‐ray characterization techniques, such as X‐ray Diffraction (XRD), Small‐Angle X‐ray Scattering (SAXS), Grazing‐Incidence Small‐Angle X‐ray Scattering (GISAXS), and Grazing‐Incidence Wide‐Angle X‐ray Scattering (GIWAXS), among others.^[^ ^22^ ^]^ For example, Kazemi et al.^[^ ^23^ ^]^ used in situ XRD under 85% relative humidity combined with liquid‐cell transmission electron microscopy to identify two simultaneous degradation mechanisms: the decomposition of CsMAFA into PbI_2_ via a dissolution/recrystallization process, and solid‐state phase segregation. However, due to the low X‐ray scattering length density (SLD) of organic compounds, it is difficult to track the evolution of these materials during degradation using XRD alone. Neutron scattering offers a solution. The distinct neutron scattering of silicon and organic materials, which can be especially triggered with deuteration (hydrogen and deuterium SLs being b_H_ = −3.74 × 10^−5^ Å, b_D_ = 6.67 × 10^−5^ Å), makes neutron scattering a powerful tool for analyzing organic layers and their degradation. This makes neutron scattering more suitable for studying organic compound degradation. Despite these advancements, such studies have primarily focused on bulk perovskite materials rather than real‐world conditions, such as degradation in thin‐film perovskite layers as found in actual devices.

Among the different neutron diffraction and small‐angle scattering techniques, neutron reflectometry provides structural information of thin films and interfaces as a function of depth along the surface or interface normal. The neutron reflectivity R, measured as a function of the wave‐vector transfer $q_{z}=\frac{4\pi }{\lambda }\sin\theta$, is modelled with 1D neutron scattering length density profiles SLD = N·b_n_ containing the element‐specific neutron scattering length density, b_n_, and the number density of the elements N in the volume considered, for example the formula unit N = 1/V(f.u.). This allows for the extraction of diffusion processes, interface roughness and density variations in the film. Although NR has been extensively used in other fields such as energy storage, polymers, and magnetism, where it provides valuable insights into the properties of interfaces and multilayered materials,^[^ ^24^, ^25^, ^26^, ^27^ ^]^ it remains relatively underutilized in the study of PSC. This is despite its potential to offer critical information about the interfaces within these devices. For example, research has shown that an excess of PbI_2_ in the perovskite composition may enhance optoelectronic processes, particularly in carrier transport and extraction.^[^ ^28^ ^]^ In line with this, Li et al.^[^ ^29^ ^]^ used time‐of‐flight polarized neutron reflectometry (TPNR) to demonstrate that a ≈ 40 nm layer of PbI_2_ at the perovskite/SiO_2_ interface can significantly reduce interfacial charge recombination, leading to improved carrier dynamics across the heterojunctions. This finding highlights the potential of NR techniques to uncover key aspects of perovskite device performance.

**Figure 1:**
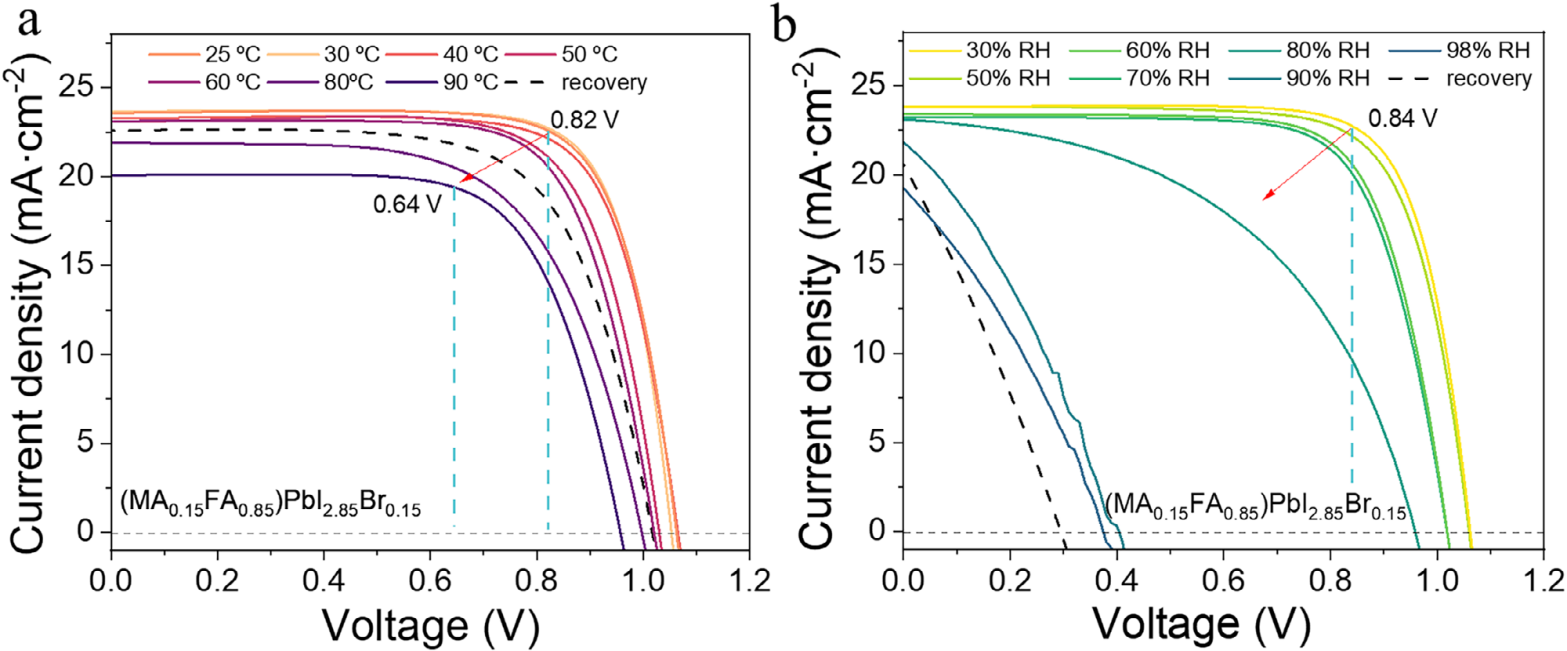
Evolution of the *J*–*V* curves during external humidity a) and thermal b) exposure (solid lines); and after external humidity and thermal exposure (dotted line) of the prepared solar cell device.

In this work, we conduct NR experiments to investigate the degradation processes caused by temperature and humidity in mixed‐cation organo‐halide PSCs. We also assess the impact of an interstitial TiO_2_ thin film on these degradation mechanisms. By incorporating this electron‐sensitive contact layer, we aim to bring the study of PSC degradation one step closer to real‐world market applications. To minimize the influence of perovskite layer roughness, we performed the reflectometry measurements with the incident beam directed from the substrate side. This approach allows for greater sensitivity to changes in the perovskite layer near the substrate interface (as shown in the top‐left part of **Figure** **1**). Specifically, the experiments provided insights into two distinct but related processes occurring in the PSC samples: i) degradation effects on both the organic cations and the inorganic structure, and ii) cation segregation within similar crystal structures. The reflectometry measurements reveal the SLD depth profile of the PSCs as degradation progresses. During this process, thin layers of the resulting compounds MABr/FAI coexist with the original (MA_0.15_FA_0.85_)PbI_2.85_Br_0.15_ PSC layer. Based on prior studies,^[^ ^28^ ^]^ we anticipate the degradation to involve the formation of layers at the interface between the perovskite and the substrate. Additionally, samples featuring a TiO_2_ layer of either 50 or 100 nm thickness, inserted between the substrate and the PSC layer, were studied using similar methods to analyse both the substrate/TiO_2_ and TiO_2_/PSC interfaces. To distinguish the degradation mechanisms associated with the two organic cations (CH_6_N and CH_5_N_2_) present in the same PSC, we deuterated one of the cations to differentiate its SLD from that of the other.

## 2 Results and Discussion

### 2.1 Photovoltaic Measurements

**Figure 2:**
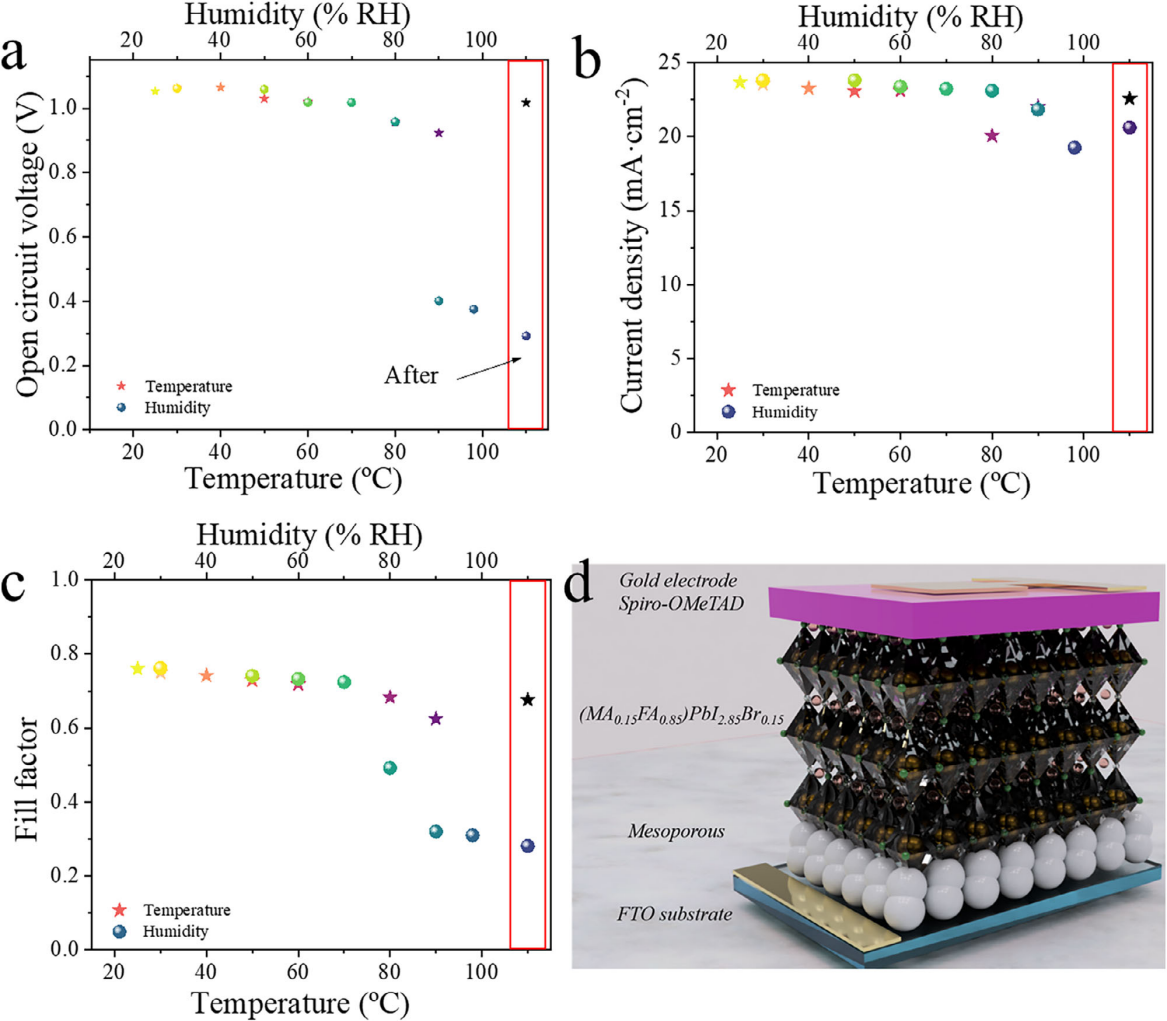
Evolution of the characteristic parameters extracted from J‐V curves during external humidity and thermal exposure. a) Open circuit voltage, b) Current density and c) Fill factor. Red rectangle is final parameters after temperature/humidity application. d) Scheme of the perovskite based solar cells used in this work.

As mentioned previously, to evaluate the effects of degradation in PSCs, we selected the optimal cell configuration in terms of efficiency and stability to fabricate the experimental devices. In previous works,^[^ ^20^ ^]^ we demonstrated the crucial role of electron‐selective contact (ESL) thickness and morphology. The choice of ESL significantly influences recombination pathways, as evidenced by variations in the ideality factor, suggesting that the electrical properties of the perovskite layer are dependent on the nature of the underlying ESL. In this study, we utilized a 100 nm‐thick mesoporous layer to infiltrate mixed perovskites such as (MA_0.15_FA_0.85_)PbI_2.85_Br_0.15_ (see scheme in **Figure** **2**d), MAPbI_3_, and MAPbBr_3_, and subjected these samples to characterization under various harsh conditions. The degradation of fully assembled devices was monitored as they were exposed to external stress factors, including high humidity (ranging from 30% to 98% RH) and elevated temperatures (25 to 90 °C). The evolution of degradation was tracked through the key photovoltaic parameters obtained from *J*–*V* curves. Figure 1 and Figure S10b (Supporting Information) present the *J*–*V* curves for the samples before (solid line) and after (dashed line) thermal or high‐humidity exposure. Notably, the temperature‐degraded samples exhibit a slight voltage drop between 0.64 and 0.82 V, which correlates with a reduction in fill factor (FF). This behaviour was consistent across all tested samples, regardless of perovskite composition, suggesting the formation of energetically localized defects, likely due to the creation of new species in the perovskite bulk or interfaces, such as PbI_2_.^[^ ^28^ ^]^ It is also observed a small drop in the current density as well as a strong drop in FF when samples are degraded with high humidity (>80% RH). The loss of the current‐voltage curve “squareness” and lower maximum power point values indicate increased non‐radiative recombination, potential phase instability or decomposition of the perovskite absorber, and degradation at the interfaces (such as the transport layers or electrode contacts). At the most extreme conditions (high humidity), the device performance is especially compromised, likely due to accelerated chemical degradation, ion migration, or moisture‐induced structural changes within the perovskite and surrounding layers. This evidence highlights the critical role of environmental stability in maintaining the performance of these devices.

To gain a better understanding of how photovoltaic parameters evolve when exposed to extreme environmental conditions, the characteristic parameters extracted from the J‐V curves (Table S1 and Figure S10a in Supporting Information) were normalized to their initial values and plotted in Figure 2 as a function of temperature and humidity. The results clearly show that all photovoltaic parameters decrease after exposure, but the extent of this reduction varies with environmental conditions. High humidity levels (≈90% RH) lead to a significant decrease in both V_OC_ and FF, while elevated temperatures (above 60 °C) primarily affect FF. When analysing the evolution of V_OC_ (Figure 2a), it was observed that exposure to low temperatures (25 to 60 °C) causes minimal V_OC_ reduction, with samples retaining more than 90% of their original value. However, as the temperature increases, V_OC_ decreases linearly, reaching ≈90% of its initial performance at 90 °C. Additionally, as humidity rises, V_OC_ follows a similar linear decline, eventually dropping to ≈40% at 90% RH. V_OC_ is governed by the balance between photogeneration and recombination processes when charge carriers are prevented from exiting the device.^[^ ^30^, ^31^ ^]^ Previous studies^[^ ^32^ ^]^ have suggested that a reduction in photogeneration may be responsible for the observed decline in V_OC_. The current density (J_SC_) remains relatively stable at moderate conditions but drops noticeably at high temperature and humidity, pointing to reduced charge carrier mobility or accelerated degradation of the active materials (Figure 2b). Figure 2c illustrates a decline in fill factor (FF) at elevated temperature and humidity, implying poorer charge extraction, increased series resistance, or interfacial losses.^[^ ^33^ ^]^ A deeper understanding of the ionic processes due to degradation by high humidity conditions has been realized in previous works.^[^ ^34^, ^35^ ^]^ These trends suggest that the primary causes of device degradation are likely related to thermal and moisture‐induced breakdown of perovskite structure, increased ionic migration, and potential delamination or decomposition at the interfaces within the multilayer device architecture depicted in Figure 2d.

In dark *J*–*V* curves (Figure S11a, Supporting Information), low shunt resistance (𝑅_𝑠ℎ_) is evidenced by elevated leakage currents, especially in the low voltage regime (near zero bias), where ideally the current should approach zero in the absence of external illumination. This enhanced recombination current results in a significant deviation from ideal diode behaviour, complicating accurate extraction of diode parameters such as the ideality factor and saturation current. In the case of temperature (Figure S11b, Supporting Information), 𝑅_𝑠ℎ_ changes drastically in the rage of 60–70 °C, which is in accordance to a drop in performance when samples are illuminated. It is observed that the three different regions A, B, C of dark current are related to shunt current, recombination and diffusion current, respectively. At last, above the built‐in potential in region D, the effect of the recombination is negligible, and the curve is determined only by the diffusion current, limited by the series resistance (R_S_) of the cell.

Therefore, two different degradation mechanisms can be suggested. In the case of humidity degradation, it is mainly the perovskite layer that suffers a chemical decomposition (drop in the shunt resistance) until there is a no return point (lack of diode behaviour). In the case of thermal degradation, it is mainly driven by a loss of electrode contacts and/or degradation of the organic hole transport layer (increment of the cell series resistance), which entail a reduction in the fill factor, although excessively high values may also reduce the short‐circuit current. It is important to highlight the self‐healing properties of the perovskite observed after the exposure to high temperature.^[^ ^36^ ^]^

### 2.2 Neutron Reflectometry

**Figure 3:**
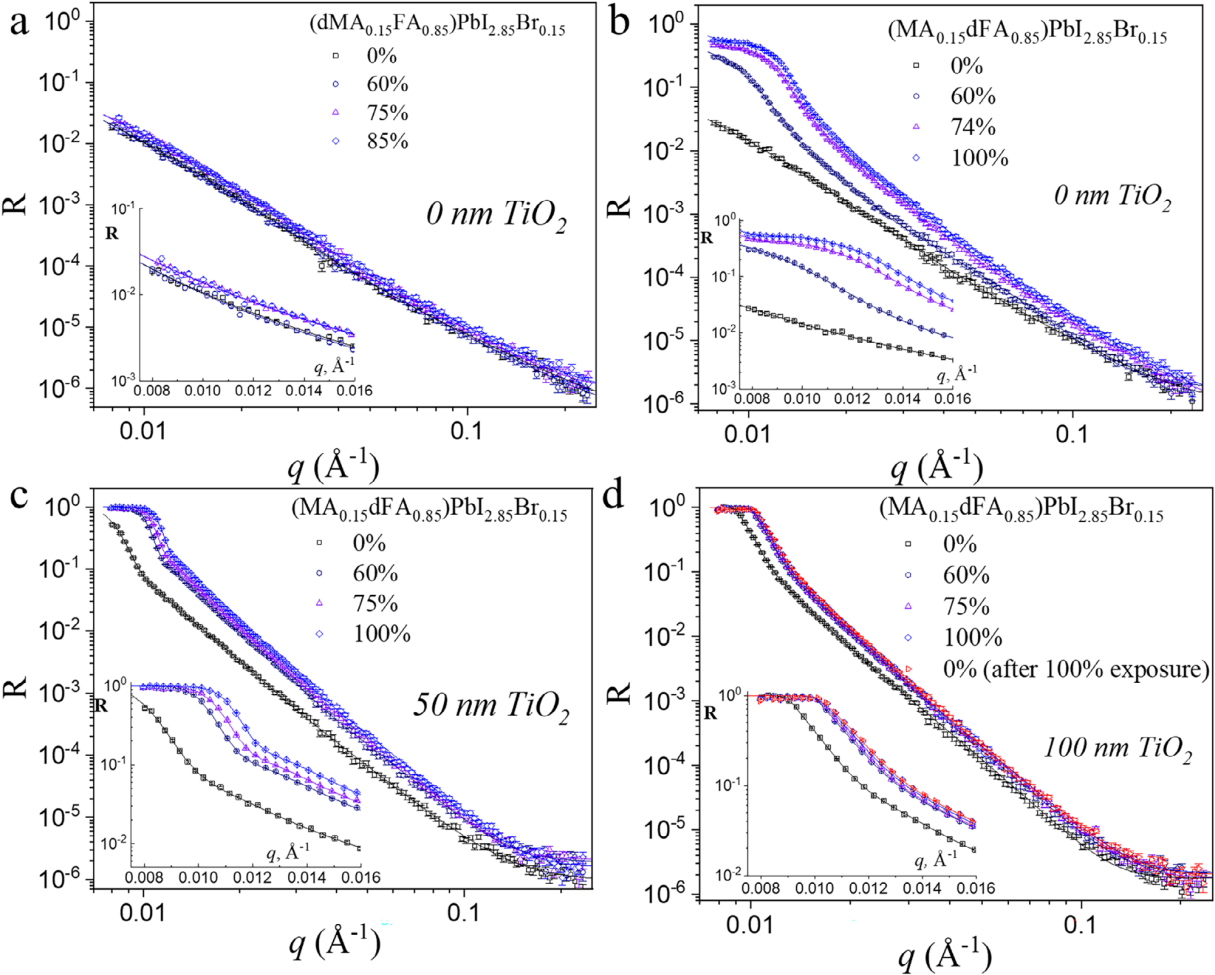
Neutron reflectivity curves for different humidities measured for a) dMA_0.15_/FA_0.85_ on Si, b) MA_0.15_/dFA_0.85_ on Si, c) MA_0.15_/dFA_0.85_ on Si/50 nm TiO_2_ and d) MA_0.15_/dFA_0.85_ on Si/100 nm TiO_2_. Insets correspond to the zoomed‐in low q regions of the curves.

**Figure 4:**
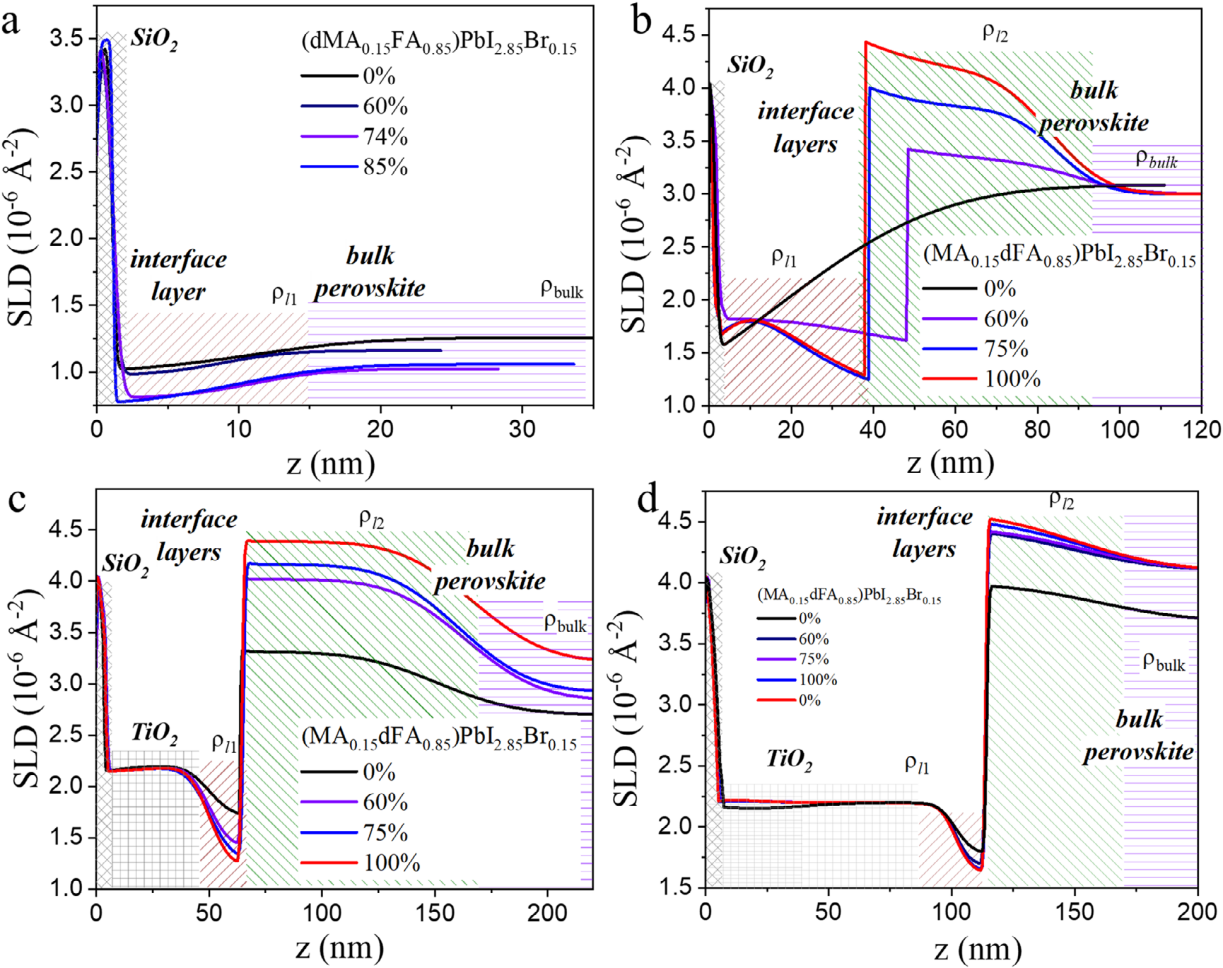
Neutron SLD profiles of the samples a) dMA_0.15_/FA_0.85_ on Si, b) MA_0.15_/dFA_0.85_ on Si, c) MA_0.15_/dFA_0.85_ on Si/50 nm TiO_2_ and d) MA_0.15_/dFA_0.85_ on Si/100 nm TiO_2_ for different humidity conditions. The respective layers are visualized and named. The thickness of the native SiO_2_ on top of the Si block presents values within the 1.5–3.0 nm range.

In situ NR experiments were performed under different temperatures (from RT up to 90 °C) and humidity levels (from 0% RH to 100% RH, using H_2_O). It should be mentioned that no qualitative changes in the NR curves with increasing temperature were detected (see Figure S12 in Supporting Information), but some dramatic changes were observed under humidity changes for some of the samples studied (**Figure** **3**; Figure S13, Supporting Information). We measured the temperature degradation (Figure S12 in Supporting Information) of (dMA_0.15_FA_0.85_)PbI_2.85_Br_0.15_, where d stands for deuterated, meaning that we deuterated one of the two organic compounds of the mixed perovskite in order to differentiate the possible degradation mechanisms of each compound, and what we observe is a slight decrease in the neutron SLD of the perovskite layer upon temperature increase. According to our models, this can be attributed to a small decomposition of the organic compounds of the perovskite, in a similar fashion to what we observe for these perovskite compositions under humidity degradation. Temperature degradation does not result in the formation of any additional layers between the substrate and the bulk perovskite. Nonetheless, as previously mentioned, the shape of the NR curves measured under humidity conditions indicates that something else may be happening as a consequence of the degradation of the perovskites as compared to the temperature degradation processes. The measured neutron reflectivity R vs scattering wave vector q curves at different humidity conditions are presented in **Figure** **4** together with the obtained fits to models of the measured samples.

For all the samples, no defined reflection fringes, typically indicative of uniform and very flat films or layers, are observed. This suggests the presence of quite rough interfaces, exceeding a few nm, between the TiO_2_, the perovskite layer and the other layers that are formed during the degradation. The absence of fringes can also be attributed to the low SLD contrast and film thickness. Nevertheless, we observe qualitative changes in R(q) from sample to sample, as well as for a specific sample with increasing humidity. Figure 3a,b shows NR curves for samples with the same nominal composition, e.g. (MA_0.15_FA_0.85_)PbI_2.85_Br_0.15_ without a TiO_2_ layer between the perovskite and the Si substrate. For the perovskite with deuterated MABr (Figure 3a), the NR curves display an absence of total reflection (R = 1 not reached), indicating a perovskite layer SLD smaller than the Si substrate SLD, and a very similar behaviour upon humidity increase, indicating the absence of dramatic changes in the behaviour of dMABr (i.e. a rather stable behaviour) when the humidity increases. For the sample with deuterated FAI (Figure 3b), a total reflection appears at low q (R = 1 at q<q_c_) when the humidity departs from 0%, a behaviour that may be attributed to the appearance of a compound with a higher amount of deuterated component. This allows us to conclude that the degradation mechanism in these perovskites is associated with a greater stability of MABr with respect to FAI.^[^ ^37^, ^38^, ^39^ ^]^ Therefore, the discussion of the subsequent NR measurements is restricted to perovskites based on deuterated FAI.

We analyse the humidity degradation processes in samples without (Figure 3b) and with the presence of a 50 nm (Figure 3c) or 100 nm (Figure 3d) thick mesoporous TiO_2_ layer between the bulk perovskite MA_0.15_/dFA_0.85_ and the substrate. The details of their structures were further revealed by fitting all the R(q) curves with models that consider the existence of two layers (l1 and l2) between the substrate (Figure 4b) or the TiO_2_ layer (Figure 3c,d) and the bulk perovskite. Just for clarification, structural models consisting of just the Si block (with a native SiO_2_ layer on top of it), the mesoporous layer (when present in the sample) and the perovskite layer were unsuccessful when fitting the data, making it obvious that additional layers, resulting from the degradation process, were required in the model in order to successfully fit the NR curves.

The SLD profiles, ρ(z), obtained from the successful fittings to the NR curves of Figure 3, are presented on Figure 4, where z corresponds to the distance from the surface of the silicon block (z = 0) toward the upper part of the bulk perovskite, crossing all the layers forming the studied samples. For the sample without TiO_2_, the model fitted to the NR curve measured from the fresh sample (0% humidity, Figure 4b) is qualitatively similar to the one obtained for the dMABr perovskite (0% humidity, Figure 4a): they present a smooth transition from the substrate (i.e. from the 1.5 nm thick SiO_2_ native oxide layer) to the bulk perovskite SLD (ρ_bulk_) through a transitional interface layer that accounts for perovskite crystal defects and/or vacancies close to the substrate.^[^ ^40^, ^41^ ^]^ Upon degradation, this interface layer is split into two well‐differentiated layers, l1 and l2, with a clear SLD contrast between them (ρ_l1_ and ρ_l2_), with the following relation: ρ_l1_ < ρ_bulk_ < ρ_l2_. From the fitting models, and by comparing the calculated SLD values for the nominal compounds forming the samples studied (supplementary materials Table S2, Supporting Information), we can conclude that the non‐deuterated organic compound MABr is mainly present in the interface layer 1 (l1) together with a small fraction of PbI_2_/PbBr_2_ and water molecules,^[^ ^42^ ^]^ whereas the deuterated organic compound dFAI is mainly present in the interface layer 2 (l2). This means that the humidity degradation induces a separation of the organic compounds from the bulk perovskite,^[^ ^42^ ^]^ which are the main components of the interface layers formed upon degradation. When comparing the different humidity% in Figure 4b, a clear transition from a smooth composition of the interface layer (0%) to the formation of l1 and l2 with well separated organic cations from the degradation together with PbI_2_/PbBr_2_ and water molecules at 60% humidity is evidenced, followed by an increase of the proportion of non‐deuterated (l1) and deuterated (l2) organic cations in these layers, revealed by the decrease (l1) and increase (l2) of their SLD values, upon 100% humidity degradation.

**Figure 5:**
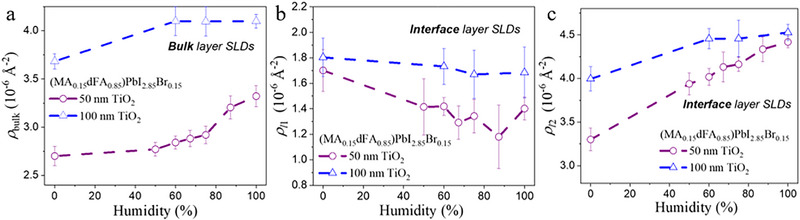
Changes of the SLD values of a) the bulk perovskite (ρ_bulk_), b) l1 interface layer (ρ_l1_) and c) l2 interface layer (ρ_l2_) of the MA_0.15_dFA_0.85_ samples with 50 and 100 nm thick TiO_2_ layers upon humidity degradation.

The SLD profiles of the humidity degradation processes of the samples with 50 nm and 100 nm thick mesoporous TiO_2_ layers are presented in Figure 4c,d, respectively. The first noticeable difference with respect to the samples without TiO_2_ is the presence of two interface layers instead of a smooth, simple one. These interface layers correspond to a lower SLD layer (l1) close to the TiO_2_ layer and a higher SLD layer (l2) close to the bulk perovskite. The model suggests that l1 consists of a layer with a higher volume fraction of non‐deuterated MABr‐based perovskite nanocrystals infiltrated into the TiO_2_ mesoporous layer, while l2 is formed by a higher volume fraction of deuterated FAI‐based perovskite nanocrystals closer to the bulk perovskite film, demonstrating the criticality of interface engineering of mesoporous layers in perovskite solar cell devices.^[^ ^43^, ^44^ ^]^ Upon humidity increase, the SLD profiles of the two sets of samples present a similar behaviour to the one explained for the perovskite without a mesoporous layer: there is an increase of the proportion of non‐deuterated (l1) and deuterated (l2) organic cations in these layers, evidencing that the degradation mechanism is similar in the three cases. Nonetheless, there are noticeable differences between the minimum (maximum) SLD values for l1 (l2) for different degrees of humidity: the sample without TiO_2_ (**Figure** **5**b) presents the largest variation in SLD values between the minimum and maximum humidity measurements (from ≈ 1.7 at 60% to ≈ 1.2 at 100% in l1, and from ≈ 3.4 at 60% to ≈ 4.5 at 100% in l2), whereas the sample with a 100 nm thick TiO_2_ layer presents a much smaller variation of SLD values (from ≈ 1.65 at 60% to ≈ 1.6 at 100% in l1, and from ≈ 4.3 at 60% to ≈ 4.5 at 100% in l2), and the 50 nm thick mesoporous layer sample presents SLD values in between these. This indicates the stronger stability upon humidity degradation of the perovskite layers in the presence of a mesoporous TiO_2_ layer, which is further increased with increasing mesoporous thicknesses.^[^ ^20^ ^]^

The l1 interfacial layers have thicknesses of ≈ 30–40 nm for the perovskite with dMA_0.15_/FA_0.85_ composition and no TiO_2_, and ≈ 10–15 nm for the other combinations of perovskite + mesoporous samples, whereas the high‐SLD l2 interfacial layers have thicknesses of ≈ 40–100 nm. The roughness, σ, of these layers was allowed to vary freely during the fit in a wide range between 0.1 and 30 nm. As can be seen on Figure 5, the interface between l1 and l2 layers always resulted in being step‐like (σ < 1.0 nm), while the other interfaces were relatively smooth, meaning that the samples present a smooth composition gradient between TiO_2_ and l1, as well as between l2 and the bulk perovskite.

The SLDs (and, thus, the compositions) of the bulk and interfacial layers change during humidity perovskite degradation, evidencing the degradation process of the samples. For the sample with MA_0.15_dFA_0.85_ on top of 100 nm TiO_2_ (Figure 4d) an additional measurement at 0% humidity after the exposition to 100% humidity (i.e. maximum humidity degradation) was also performed, evidencing that the composition of the layers did not recover, in agreement with the *J*–*V* measurements and indicating the irreversible character of PSC degradation under humidity.

To further visualize the changes in the samples upon degradation, the evolution of the SLD of the different layers forming the MA_0.15_dFA_0.85_ samples with 50 and 100 nm thick mesoporous layers as a function of the humidity% is presented in Figure 5. For the bulk perovskite (Figure 5a), an increase in the SLD with the humidity% is observed. This can be attributed to the degradation of the perovskite, which separates its organic and inorganic parts, resulting in a bulk perovskite with a higher volume fraction of deuterated organic compounds (dFAI) and less inorganic compounds (PbI_2_, PbBr_2_), which go to the interface layer, as previously discussed. The SLD changes of the first interface layer, l1, and of the second one, l2, upon humidity exposure are shown in Figure 5b,c, respectively. The SLD decrease of the l1 layer and the increase of the SLD of layer l2 may be attributed to the higher volume fraction of MABr and water molecules with respect to PbI_2_/PbBr_2_ in l1, and to the increase in dFAI content in l2, respectively, upon humidity increase, as explained previously.

Overall, the obtained NR results show improved structural stability of the perovskite layers coated on top of TiO_2_ mesoporous nanolayers, both in the structure of the fresh samples (i.e. measurements with 0% humidity) and after humidity degradation. The humidity degradation process yields the formation of two interstitial layers between the perovskite and the silicon or mesoporous layers: l1, composed of MABR_2_, PbI_2_/PbBr_2_ and water molecules, with thicknesses between 30 and 40 nm; and l2, mostly composed of deuterated FAI, with thicknesses between 40 and 100 nm, as compared to the >500 nm thick bulk perovskite layers. This is in good agreement with previous proposed perovskite degradation routes.^[^ ^45^ ^]^These findings can also explain the lack of performance of the full perovskite devices (Figures 1 and 2), when they are exposed to high humidity.^[^ ^46^ ^]^

### 2.3 MD Simulation of the Slab Degradation

To support our findings on the degradation of (MA/FA)Pb(Br/I)_3_ under varying humidity and temperature, we conducted classical MD simulations, as detailed in the methodology section. Simulating 20‐100% RH proved challenging: low water vapor concentrations provide a few water molecules for interactions with the perovskite slab, preventing statistically meaningful degradation events during the MD simulations' timescales.

**Figure 6:**
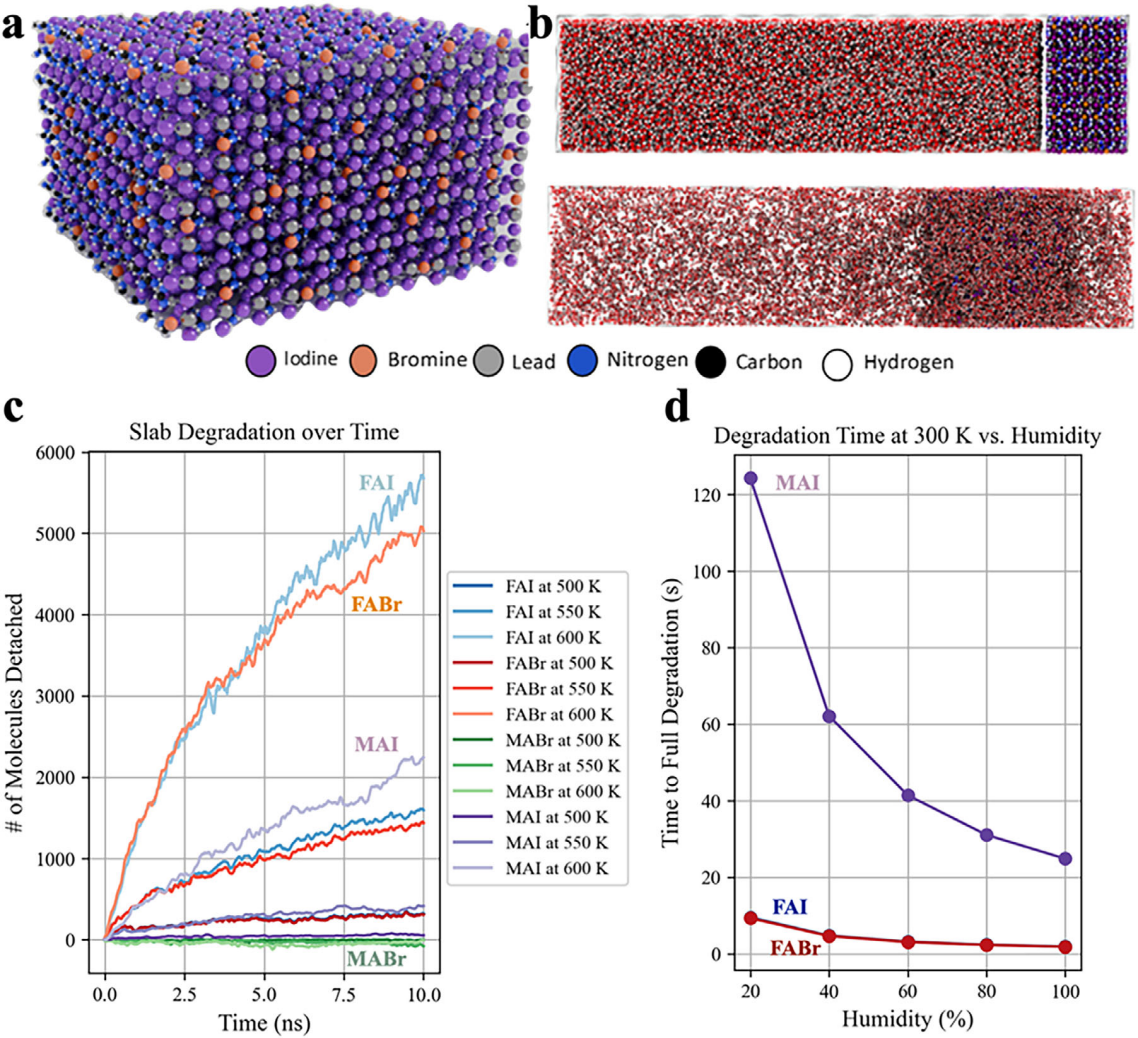
a) The Slab model used in the simulation (see parameters' Table S4, Supporting Information). b) Simulation box shown at t = 0 ns (up) and at t = 100 ns (down), evidencing the water infiltration in the perovskite after the MD simulation is initiated. c) Organic clusters at different temperatures, d) with the necessary time for a full degradation of the slab at 300K.

To overcome these constraints, we instead simulated the slab in bulk water (**Figure** **6**a), accelerating degradation events for detailed analysis and extrapolation to different humidity and temperature conditions. The degradation was modelled with a first‐order kinetic model, expressing rates as a function of theseriables, which can be extrapolated to real‐world scenarios. This process is illustrated in Figure 6b, where the upper panel corresponds to the initial state of the simulation (t = 0), showing a well‐defined interface between the bulk perovskite material (right side of the simulation box) and the water (left side), while the lower panel corresponds to t = 100 ns, in which the infiltration of water molecules into the perovskite is clearly observed.

**Figure 7:**
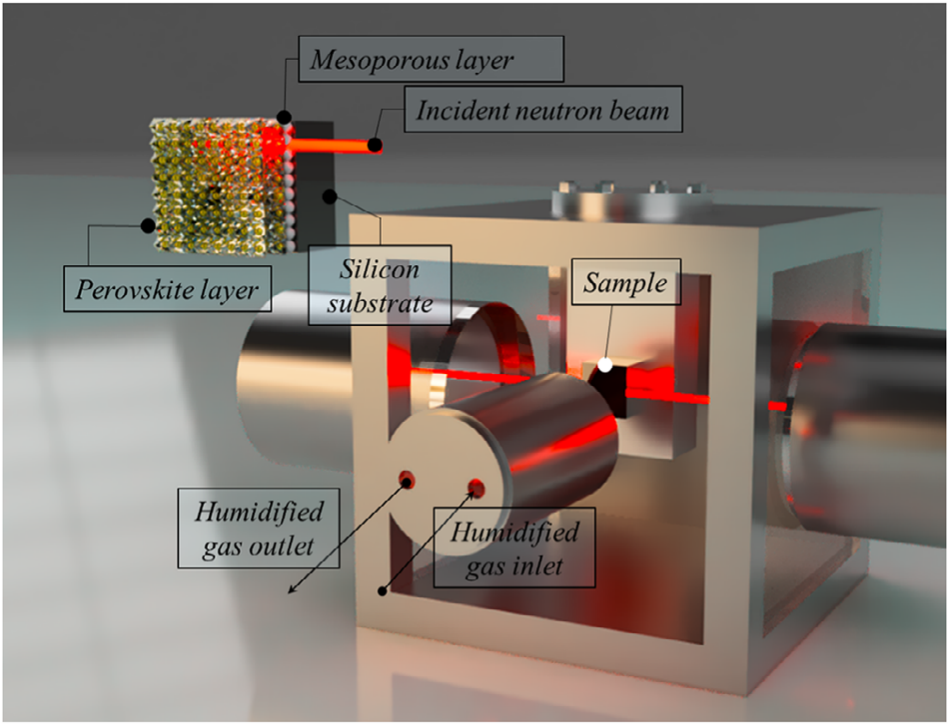
Schematic view of the humidity sample chamber for in situ NR measurements. Zoom‐in of the sample configuration and the position of the incident neutron beam (top left).

The degradation kinetics over time were modelled as: N(t)_detached_ =  N_0_ (1 − e^−kt^), where N(t)_detached_ represents the number of detached molecules at time t, N_0_ is the initial number of molecules, and k denotes the degradation rate. **Figure** **7**c,d displays the degradation of ionic pairs – FAI, FABr, MAI and MABr – revealing rapid desorption for FAI and FABr, a slower process for MAI, and virtually no desorption for MABr, due to its limited presence in the lattice. Using these plots, we determined rates for each molecular species and estimated activation energy for desorption via the Arrhenius equation: $k=A\;e^{-\frac{E_{a}}{\mathrm{RT}}}$, where k is the detachment rate, A is the pre‐exponential factor, E_a_ is the activation energy (kcal mol^−1^), R is the gas constant (8.314 J mol^−1^ K^−1^), and T is the temperature in Kelvin. With these rates, we estimated perovskite degradation times under various humidity conditions (see Table S3, Supporting Information), showing rapid desorption of FAI and FABr—fully eliminated within seconds, accelerating with higher humidity—while MAI degraded more slowly, though also faster at elevated humidity. Despite limitations from approximations (e.g. first‐order kinetic model, high‐temperature extrapolation), the qualitative trends align well with experimental data. Desorption of FA‐based ion pairs (with Br or I) occurs swiftly, on seconds timescale, whereas MA‐based ion pairs degrade more gradually, with MABr appearing largely unaffected. Given the lower MA content in the lattice compared to FA, the weaker MA degradation signal observed in neutron reflectometry experiments is consistent with these findings.

## 3 Conclusion

Neutron reflectometry measurements provided valuable insights into the structural changes occurring within the hybrid perovskite films. One of the key observations was the impact of the TiO_2_ layer on the stability of the structural layers within the solar cell. TiO_2_, commonly used as an electron transport layer, plays a crucial role in enhancing charge transport and minimizing recombination losses. However, this study revealed that the presence of the TiO_2_ layer could also affect the overall stability of the perovskite structure, suggesting a complex interplay between the transport layers and the active material. Therefore, understanding this interaction is essential for optimizing the architecture of perovskite solar cells and enhancing their long‐term stability.

Another critical finding was the weak structural changes observed in the perovskite films as the temperature increased to 150 °C. This indicates a certain level of thermal stability within the hybrid perovskite materials, suggesting that they can withstand elevated temperatures to some extent without significant degradation. However, this thermal resilience must be balanced against other environmental factors that can compromise the material's integrity.

In contrast, the effects of humidity on the structural stability of the perovskite films were found to be much more pronounced. The study demonstrated stronger changes in the perovskite film structure when exposed to humidity levels of up to 100%. This finding is particularly concerning, as high humidity can lead to hydrolysis reactions that can degrade the perovskite material, resulting in the formation of unwanted byproducts that may impede charge transport and overall device performance.

Furthermore, the study shed light on the changes occurring at the interfacial layers of the perovskite films. Up to our knowledge, this is the first study that proves the degradation mechanism in complex mixed cation PSCs by neutron reflectometry in thin films. It was observed that there is an accumulation of FAI within a 50–100 nm layer near the surface, alongside an accumulation of MABr in a thinner layer at the bottom of the film. This stratification at the interfaces can significantly affect charge transport properties and overall device performance. The accumulation of FAI suggests that these materials may migrate toward the surface, potentially enhancing stability at the surface while compromising the bulk properties. Conversely, the presence of PbI_2_ at the bottom of the bulk perovskite film may act as a charge trapping layer, leading to increased recombination losses and decreased efficiency.

In summary, this study contributes to the understanding of the stability and degradation mechanisms of hybrid perovskites, providing critical insights into the effects of temperature, humidity, and layer interactions on structural integrity. The confirmed degradation observed through *I*–*V* measurements highlights the need for further research into improving the long‐term stability of perovskite solar cells. The findings related to the TiO_2_ layer's impact on structural stability underscore the importance of optimizing layer architectures to enhance performance. Moreover, the pronounced effects of humidity on structural changes raise concerns about the environmental resilience of hybrid perovskites, indicating that effective encapsulation methods are essential for protecting these materials from moisture‐induced degradation. Lastly, the examination of interfacial layer dynamics reveals complex material behaviour that can influence overall device efficiency. By elucidating the degradation pathways and environmental sensitivities of hybrid perovskites, this work lays the groundwork for developing advanced materials and device architectures that can withstand real‐world operating conditions while maintaining optimal performance.

## 4 Experimental Section

### Materials

Lead iodide PbI_2_ (99.8%) (from Tokyo Chemical Industry (TCI)) was used without further purification. TiO_2_ nanoparticles (30 NRD), methylammonium iodide (>99.99%) (***MAI***), methylammonium bromide (>99.99%) (***MABr***) and formamidininium iodide (>99.99%) (***FAI***) were obtained from Greatcellsolar materials and 2,2´,7,7´‐tetrakis(N, N‐di‐p‐methoxyphenyamine)‐9,9‐spirobifluorene (Spiro‐OMeTAD) 99% (HPLC) was acquired from Merck KGaA. Cyanamide (Sigma–Aldrich, 99%), acetic acid (Sigma–Aldrich, ≥99.8%), palladium on charcoal (Fluka, 10% Pd), deuterium gas (Linde, 99.8 vol% D), methylamine‐d3 (Sigma–Aldrich, 99.9% D), hydriodic acid (Sigma–Aldrich, 99.99%, 57 wt% HI), hydrogen bromide solution (Sigma–Aldrich, 33 wt% in acetic acid), methanol (VWR ≥99.8%), ethanol (Merck, ≥99.2%), diethyl ether (VWR, ≥99.7%), dichloromethane (Sigma–Aldrich, ≥99.8%) and deuterium oxide (Sigma–Aldrich, 99.9% D) were used as received.

i) *Synthesis of formamidinium iodide‐d5, methylammonium iodide‐d6 and methylammonium bromide‐d6*.

### Materials—NMR Characterization

1H‐, 2H‐ 13C‐NMR spectra were recorded on a Bruker Avance HD III 600 mHz spectrometer equipped with a BBO Prodigy cryo‐probe. Samples were measured at 295 K in DMSO‐d6 or DMSO‐h6. (Figures S1–S9, Supporting Information)

ii) *Synthesis of formamidinium iodide‐d5 (* ***dFAI***)

### Materials—Formamidinium Acetate‐d1


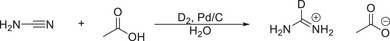


The reaction was done similarly to the literature procedures.^[^ ^47^, ^48^ ^]^ 0.140 g of palladium on charcoal was suspended in 8 ml of acetic acid and 42.5 ml of water. Deuterium gas was added via a glass frit (porosity 2) under stirring for 30 min. 4.986 g of cyanamide was dissolved in 50 ml of water and added to the mixture. Deuterium gas was added for seven hours (≈5 bar), and the reaction was stirred at room temperature. The progress of the reaction was checked by adding a droplet of the reaction mixture to a few millilitres of a solution of 1 m silver nitrate in 10% ammonia in water. A yellow precipitate showed the presence of cyanamide. After, no cyanamide could be detected by this method, and NMR measurements confirmed the completion of the reaction. The mixture was poured through a folded filter. The catalyst was washed with water, and all the water was removed at a rotary evaporator. The product was dried in the vacuum oven overnight, yielding 9.942 g of formamidinium acetate‐d1 (79.8%).

### Materials—Formamidinium Iodide‐d1


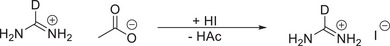


The reaction was done similarly to a literature procedure.^[^ ^49^ ^]^ 3.18 g of formamidinium acetate‐d1 was dissolved in 20 ml of methanol. Under stirring in an ice bath, 8.421 g of hydriodic acid was added dropwise over ten minutes. After stirring for two hours at room temperature, the methanol was removed at a rotary evaporator. The brown solid material was washed with diethyl ether and dried in the vacuum oven over night, yielding 5.134 g of formamidinium iodide‐d1 (98.0%).

### Materials—Formamidinium Iodide‐d5

The reaction was done similarly to a literature procedure.^[^ ^50^ ^]^ To remove last traces of iodine, 4.964 g of formamidinium iodide‐d1 was recrystallized from ethanol and dried in the vacuum oven overnight, yielding 2.34 g of which 0.962 g were mixed with 35 g of deuterium oxide.

The deuteration degree of C‐D was 99.9% (manufacturer information). The deuteration degree of N‐D was calculated from the 2H‐NMR in DMSO‐h6 by comparison with the CD3 intensity to be 95.3%.

iii) *Synthesis of methylammonium iodide‐d6 (* ***dMAI***)

### Materials—Methylammonium Iodide‐d3


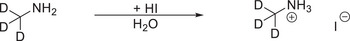


The reaction was done similarly to a literature procedure.^[^ ^51^ ^]^ 2.397 g of methylamine‐d3 was distilled into a Schlenk flask and then transferred into a two‐neck flask with 9.617 g of water via high vacuum distillation. The flask was stirred in an ice bath, and hydriodic acid was added dropwise in 30 min. After stirring at room temperature overnight, the solution was transferred (with partial loss) into another flask and water was removed at a rotary evaporator. The solid product was recrystallized from ethanol, washed with dichloromethane and dried in the vacuum oven overnight, yielding 3.101 g of slightly yellow crystals of methylammonium iodide‐d3 (27.2%).

### Materials—Methylammonium Iodide‐d6

The reaction was done similarly to a literature procedure.^[^ ^51^ ^]^ 2.935 g of methylammonium iodide‐d3 was mixed with 125 g of deuterium oxide and stirred overnight at room temperature. After removal of the deuterium oxide at a rotary evaporator, the product was dried under high vacuum, yielding 2.928 g of methylammonium iodide‐d6 as a white solid (97.9%).

The deuteration degree of C‐D was 99.9% (manufacturer information). The deuteration degree of N‐D was calculated from the 2H‐NMR in DMSO‐h6 by comparison with the CD3 intensity to be 98.0%.

iv) *Synthesis of methylammonium bromide‐d6 (* ***dMABr***)

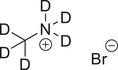


### Materials—Methylammonium Bromide‐d3

The synthesis of methylammonium bromide‐d3 was done according to the procedures for methylammonium iodide‐d3 using 2.543 g of methylamine‐d3 and 18.495 g of hydrogen bromide solution. Finally, 4.676 g of product was obtained (54.5%).

### Materials—Methylammonium Bromide‐d6

The synthesis of methylammonium bromide‐d6 was done according to the procedures for methylammonium iodide‐d6 using 4.559 g methylammonium iodide‐d3 and 317 g of deuterium oxide. 4.585 g of product was obtained (98.0%).

The deuteration degree of C‐D was 99.9% (manufacturer information). The deuteration degree of N‐D was calculated from the 2H‐NMR in DMSO‐h6 by comparison with the CD3 intensity to be 95.3%.

### Methods—Solar Cell Device Fabrication

Perovskite‐based solar cells were fabricated following a n‐i‐p configuration. For that, an FTO (NSG10) substrates were cleaned by a sequential treatment in the ultrasound bath in a 2% Hellmanex solution (in water) and isopropanol followed by ozone treatment for 20 min. The substrates were then heated to 500 °C, and a compact blocking layer of TiO_2_ was deposited by spray pyrolysis using a diluted 1:19 mL of titanium(IV) diisopropoxide bis(acetylacetonate) solution in ethanol. Once the samples were cooled down to room temperature, a mesoporous dispersion of TiO_2_ nanoparticles (30 NRD) in ethanol (1:7) was spin‐coated at 4000 rpm for 30 s, followed by a progressive heating step till 500 °C for 30 min to obtain the 50 and 100 nm mesoporous layer thickness, respectively.^[^ ^52^, ^53^ ^]^ Stoichiometric precursor solutions (1 m) were prepared by mixing (MABr_0.15_/FAI_0.85_) with PbI_2_ in N, N´‐dimethylsulfoxide (DMSO) and kept under stirring at 70 °C overnight in order to dissolve PbI_2_ completely. The perovskite layers were fabricated using a two‐step spin‐coating process following previous work.^[^ ^54^ ^]^ To deposit the hole transporting material (HTM), Spiro‐OMeTAD was spin‐coated at 4000 rpm for 30 s by dissolving 72.3 mg of Spiro‐OMeTAD in 1 mL of chlorobenzene together with 28.8 mL of 4‐tert‐butylpyridine as dopant. In order to avoid any possible contamination or degradation, the perovskite and HTM films were prepared inside an argon‐filled glove box under moisture and oxygen‐controlled conditions. Finally, 100 nm of gold was deposited by thermal evaporation.

v) *Sample preparation for neutron reflectometry experiments*

For the deposition of the perovskite and the mesoporous layers, samples have been prepared using the aforementioned protocol but using a silicon block acting as a substrate (single crystal Si block with 25 × 25 × 10 mm^3^ one side polished with roughness ≈5 Å bought from Photon Export (Av. de Cornellà, 128, 08950 Esplugues de Llobregat, Barcelona, Spain).

### Methods—Neutron Reflectometry Experiments

NR measurements were carried out initially at the D17 beamline of the Institute Laue‐Langevin (Grenoble, France),^[^ ^55^, ^56^ ^]^ and subsequently at the time‐of‐flight Spatz reflectometer of the 20 mW OPAL reactor of the ACNS (ANSTO, Australia).^[^ ^57^ ^]^ The sample was placed on a vertical plate within an environmental chamber on the sample stage (Figure 7). To control the relative humidity in the sample environment, a Hiden Isochema XCS dynamic vapor delivery system controlled using HIsorp Systems Software (Hidden Isochema Ltd., Warrington, UK) was used. Very stable values of 0, 60, 75 and 85% RH using H_2_O vapor were achieved during the measurement. Two different neutron incident angles (0.7 ° and 3.0 ° using a 20 mm footprint) were utilized, and combined the reflectivity curves from the two angles to generate the final data to cover the entire range of *q* studied (0.008–0.24 Å^−1^). Reflectivity curves were measured with a ΔQ/Q resolution ≈5%. The NR data were fitted by a number of structural models using the RefnX package to minimize the difference between the experimental and theoretical curves.^[^ ^58^ ^]^ The estimates of obtained parameters deviations were made using Markov chain Monte Carlo algorithms (MCMC sampling).^[^ ^58^, ^59^ ^]^ This also allowed to analyse the likelihood of the models of the considered structures.

### Methods—Degradation Characterization of Full Devices

Current–voltage curves were measured inside a climate chamber (BINDER™ 9020‐0407), varying the temperature (30 to 90 °C) and humidity (10 to 98%RH) conditions with a potentiostat (Keithley 2604). *J*–*V* measurements were performed under 1 sun illumination at 100 mV s^−1^ scan rate (pre‐sweep delay: 10 s) using a black metal mask (0.56 cm^2^) over the square solar cell active area to reduce the influence of scattered light.

### Methods—Statistical Analysis

Statistical analyses were performed using Microsoft Excel. No data transformation or outlier exclusion was applied prior to statistical testing. Data were presented as mean ± standard deviation (SD) or mean ± standard error of the mean (SEM), as indicated in figure legends. A minimum of five samples (n ≥5) were used for each experiment unless otherwise specified.

### Methods—Computational Methods

i) *Slab Models and Boxes*

To construct the slab model, a charge‐balanced (MA/FA)Pb(Br/I)_3_ unit cell was begun with, and replicated it in a 2 × 2 × 2 along each axis. Along the *z*‐axis, the (001) facet was exposed, defining the finite direction. The slab was cut to terminate with the AX layer (A = MA/FA; X = Br,I) on both sides. Atomic positions in the slab were relaxed at the density functional theory (DFT) level, using the PBE^[^ ^60^ ^]^ exchange‐correlation functional and a DZVP basis‐set, with effective core‐potentials applied on all atoms. These calculations were run using the CP2K^[^ ^61^ ^]^ 2024.1 package. Following the slab setup, we carried out an ab initio molecular dynamics simulation (AIMD). First, an equilibration step was conducted in an NVT ensemble for 2 picoseconds with a 1 fs timestep. This was followed by an NPT ensemble simulation for 5 picoseconds with a 1 fs timestep, allowing the cell parameters in the x and y directions to relax, facilitated by the analytical stress tensor implemented in CP2K. From the AIMD, we computed radial distribution functions for all atomic pairs, which were subsequently used to parametrize a classical force‐field potential via the auto‐FOX^[^ ^62^, ^63^ ^]^ packages developed by some of us. In this force‐field, interactions among the inorganic ions and between the inorganic ions and the organic species were modelled with point charges using Coulomb electrostatics and Lennerd‐Jones potentials for non‐bonding interactions. Charge values and Lennard‐Jones sigma parameters were optimized by fitting DFT‐derived radial distribution functions to those obtained with the classical force‐field. Intramolecular interactions within the organic cations, FA and MA, included bonded contributions.

For the classical molecular dynamics simulations – enabled by the force‐field above and allowing us to model a larger system than feasible with DFT to analyse the humidity‐induced degradation, we created an expanded supercell by replicating the DFT slab in a 2 × 2 × 4 arrangement. Along the z‐direction, the finite part was replicated four times, resulting in a final structure with the formula MA_176_FA_962_Pb_1008_Br_288_I_2866_, which exposes the (MA/FA)(Br/I) surface. We used the Packmol^[^ ^64^ ^]^ package to construct a 7.8 × 7.8 × 37 nm^3^ tetragonal simulation box, positioning the slab at the bottom of the box, filling the upper portion with water molecules (see Figure 7 for details about the slab and the main text for further insights).

ii) *Simulation Details*

For the molecular dynamics (MD) simulations, an initial 5 ns canonical (NVT) equilibration was performed at temperatures ranging from 300 to 600 K, in increments of 50 K, at a constant volume. This NVT phase employed an integration timestep of 1 fs. Following this, a further 2 ns equilibration was conducted under isothermal‐isobaric (NPT) conditions, with a constant pressure of 1 atm and varying temperatures. Throughout these equilibration stages at the NVT and NPT level, the positions of the slab atoms were constrained to allow for a controlled relaxation of the system. Finally, a 10 ns production MD simulation was performed, during which all constraints were removed to observe the system's behavior under fully dynamic conditions. All simulations were conducted using GROMACS version 2023.1.^[^ ^65^, ^66^, ^67^, ^68^, ^69^, ^70^, ^71^, ^72^ ^]^ The force field (FF) parameters for the slab were developed using the auto‐FOX package. Smooth Particle Mesh Ewald (SPME) and beta‐Euler splines^[^ ^73^ ^]^ were employed for long‐range interactions, and the system temperature and pressure were controlled using a velocity‐rescaling thermostat^[^ ^74^ ^]^ and the Parrinello‐Rahman barostat,^[^ ^75^ ^]^ respectively. A 1 nm short‐range cutoff was applied to compute both Lennard‐Jones and Coulomb interactions. The force field parameters for MA and FA were generated using MATCH,^[^ ^76^ ^]^ which automates the assignment of CHARMM‐based atom types and FF parameters by comparison with a dataset of chemical fragments.^[^ ^77^ ^]^ Complete parameter details are provided in the Supplementary Information (SI).

## Conflict of Interest

The authors declare no conflict of interest.

## Supporting information



Supporting Information

## Data Availability

The data that support the findings of this study are available from the corresponding author upon reasonable request.
