# Supplementary material for: Unravelling Mixed Organic‐Halide Perovskite Degradation Under Extrinsic Factors
Source: Small. 2025 Dec 8;22(6):e09525. doi: 10.1002/smll.202509525 (PMC12837359; doi:10.1002/smll.202509525)
Supplement: Supplementary file 1 — Supporting Information [file SMLL-22-e09525-s001.docx]

Supporting information

Unravelling mixed organic-halide perovskite degradation under extrinsic factors

Manuel Salado ^1,2,^*, Timur Tropin^1^, Abdessamad El Adel^1^, Lisa Sarah Fruhner^3^, Julia Bodon^4^, J.L Vilas ^1,4^, Anton P. Le Brun^5^, Thomas Saerbeck^6^, Ivan Infante^1,2^, Viktor Petrenko ^1,2^ and Jose M. Porro^1,2^.

^1^ BCMaterials, Basque Center for Materials, Applications and Nanostructures, Bld. Martina Casiano, UPV/EHU Science Park, Barrio Sarriena, s/n, 48940 Leioa, Spain;

^2^ IKERBASQUE, Basque Foundation for Science, 48013 Bilbao, Spain

^3^ Jülich Centre for Neutron Science (JCNS-1) and Institute for Complex Systems (ICS-1), Forschungszentrum Jülich GmbH, Leo-Brandt-Straße, 52425 Jülich, Germany.

^4^ Macromolecular Chemistry Group (LABQUIMAC), Department of Physical Chemistry, Faculty of Science and Technology, University of the Basque Country (UPV/EHU), Barrio Sarriena s/n, E-48940, Leioa, Spain

^5^ Australian Centre for Neutron Scattering, Australian Nuclear Science and Technology Organisation, Lucas Heights, NSW, Australia

^6^ Institut Laue-Langevin, 71 Avenue des Martyrs, Grenoble Cedex 9 38042, France

**
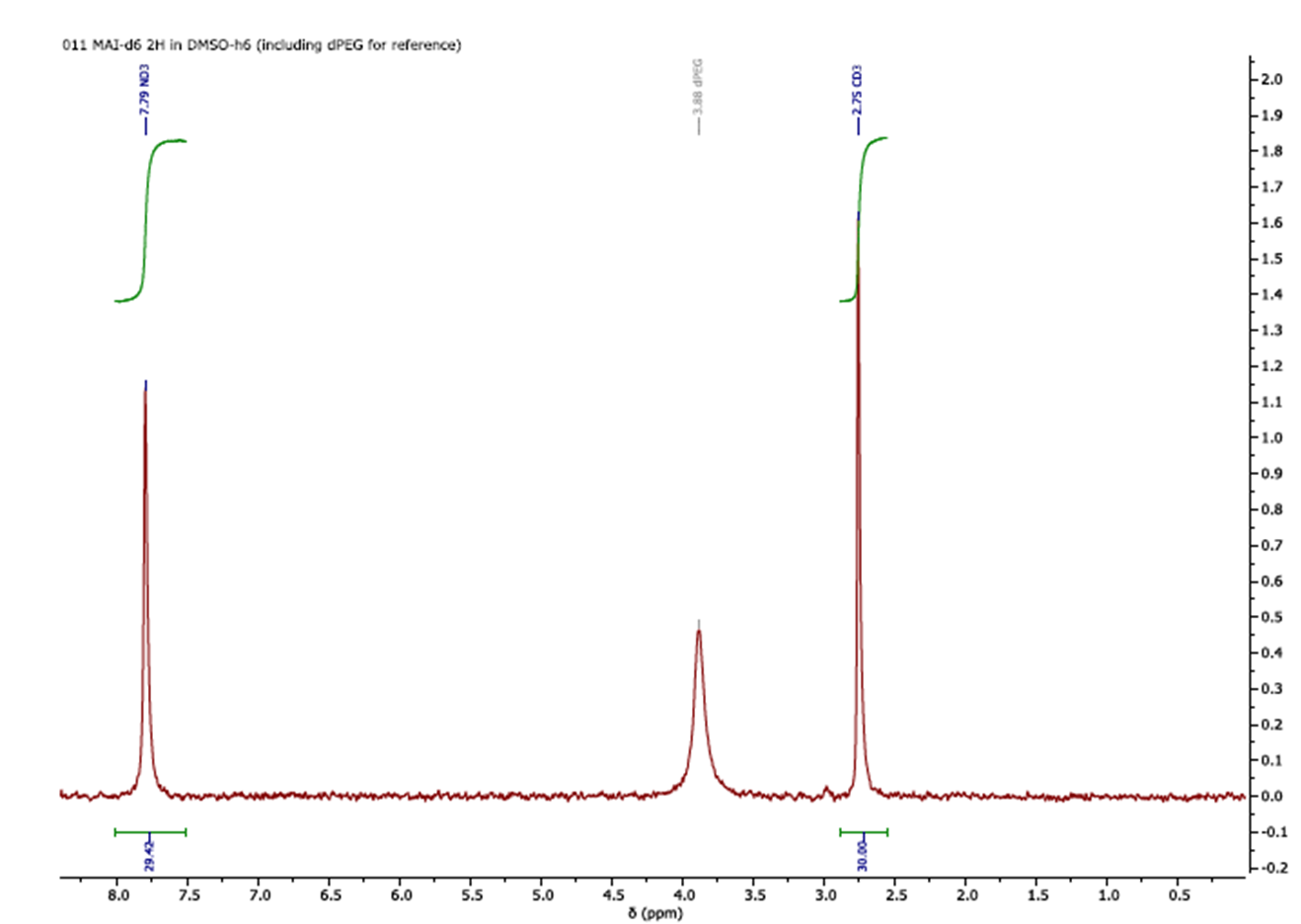
**

**Figure S1.** Static solid-state ^2^H NMR spectrum of MAI-d6 in DMSO-h6 collected at -20 °C.

**
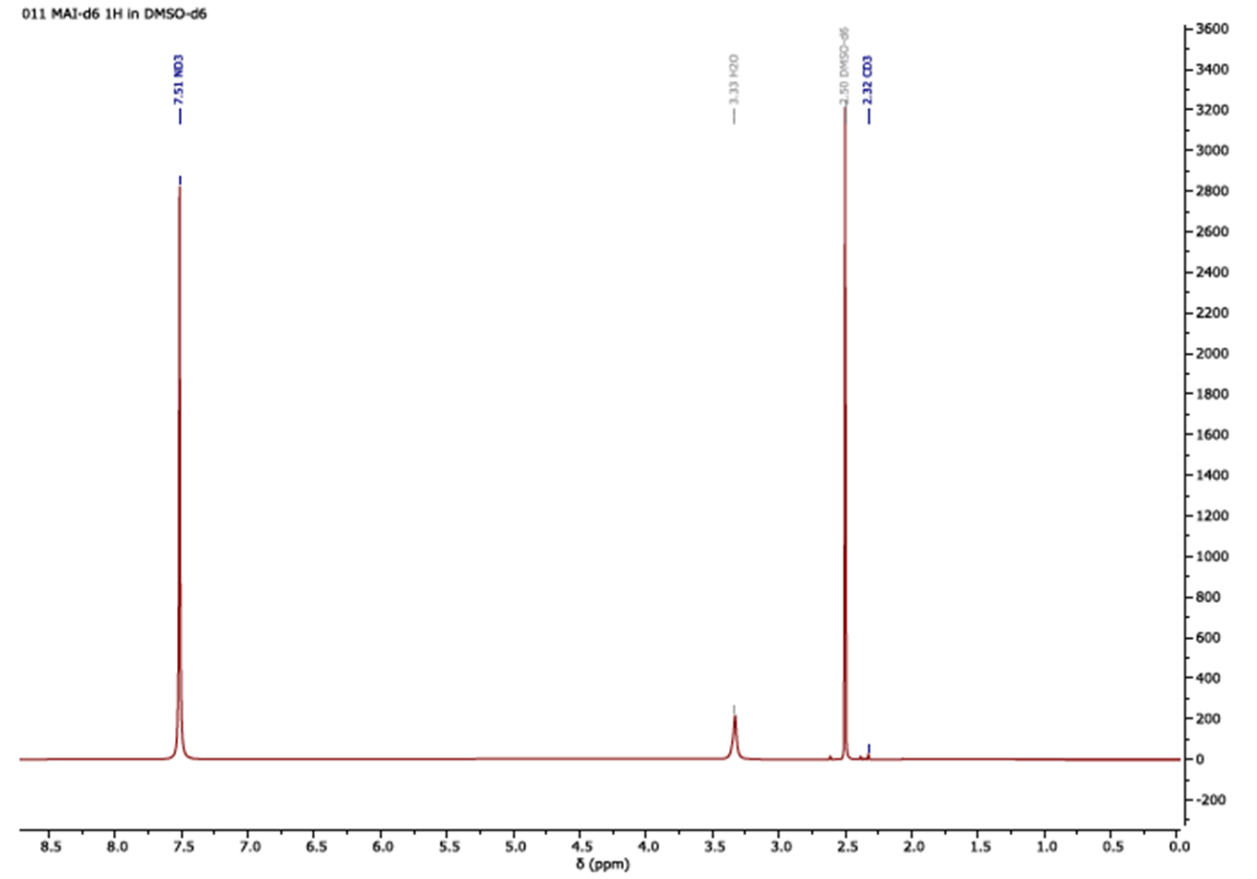
**

**Figure S2.** Static solid-state ^1^H NMR spectrum of MAI-d6 in DMSO-d6 collected at -20 °C.

**
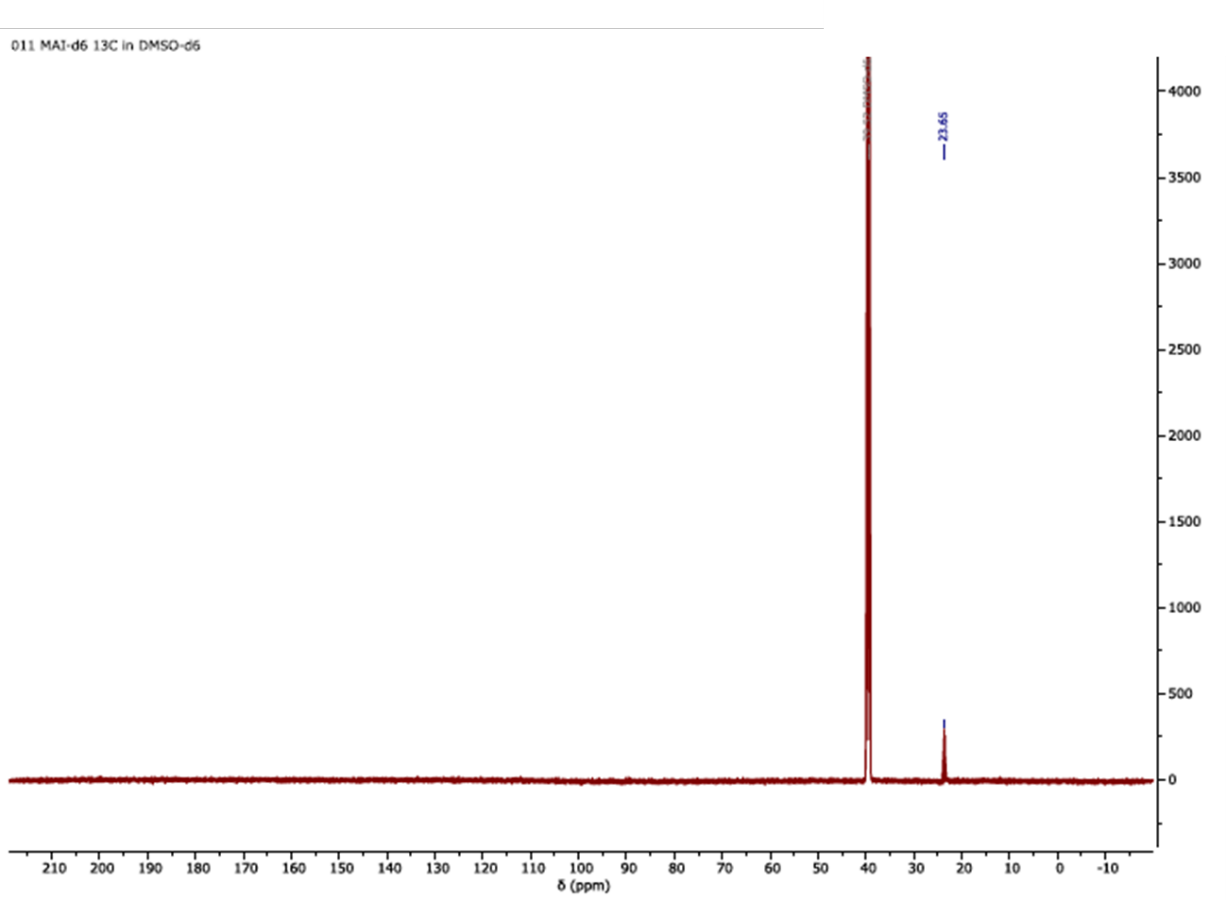
**

**Figure S3.** Static solid-state ^13^C NMR spectrum of MAI-d6 in DMSO-d6 collected at -20 °C.

**
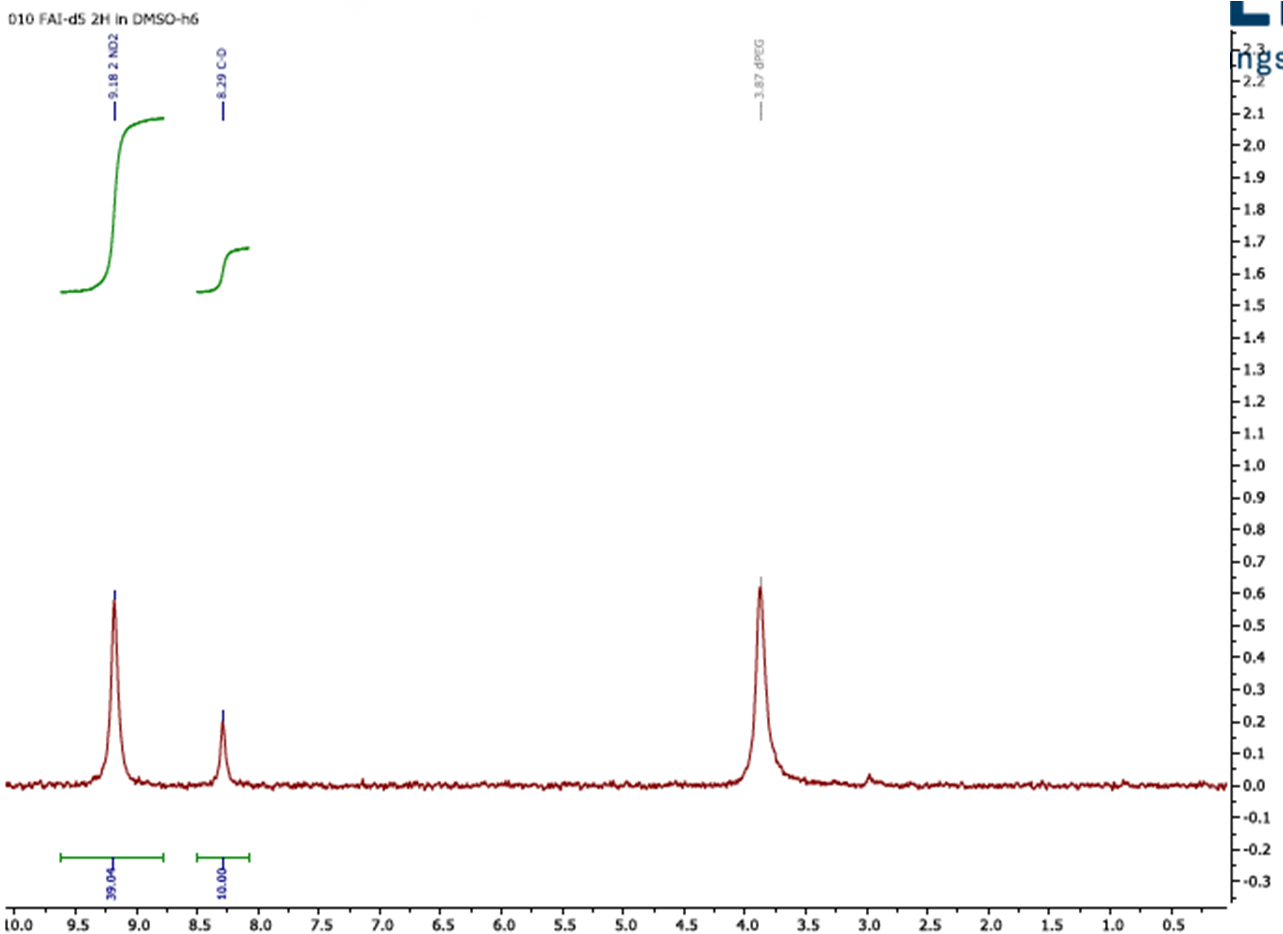
**

**Figure S4.** Static solid-state ^2^H NMR spectrum of FAI-d5 in DMSO-h6 collected at -20 °C.

**
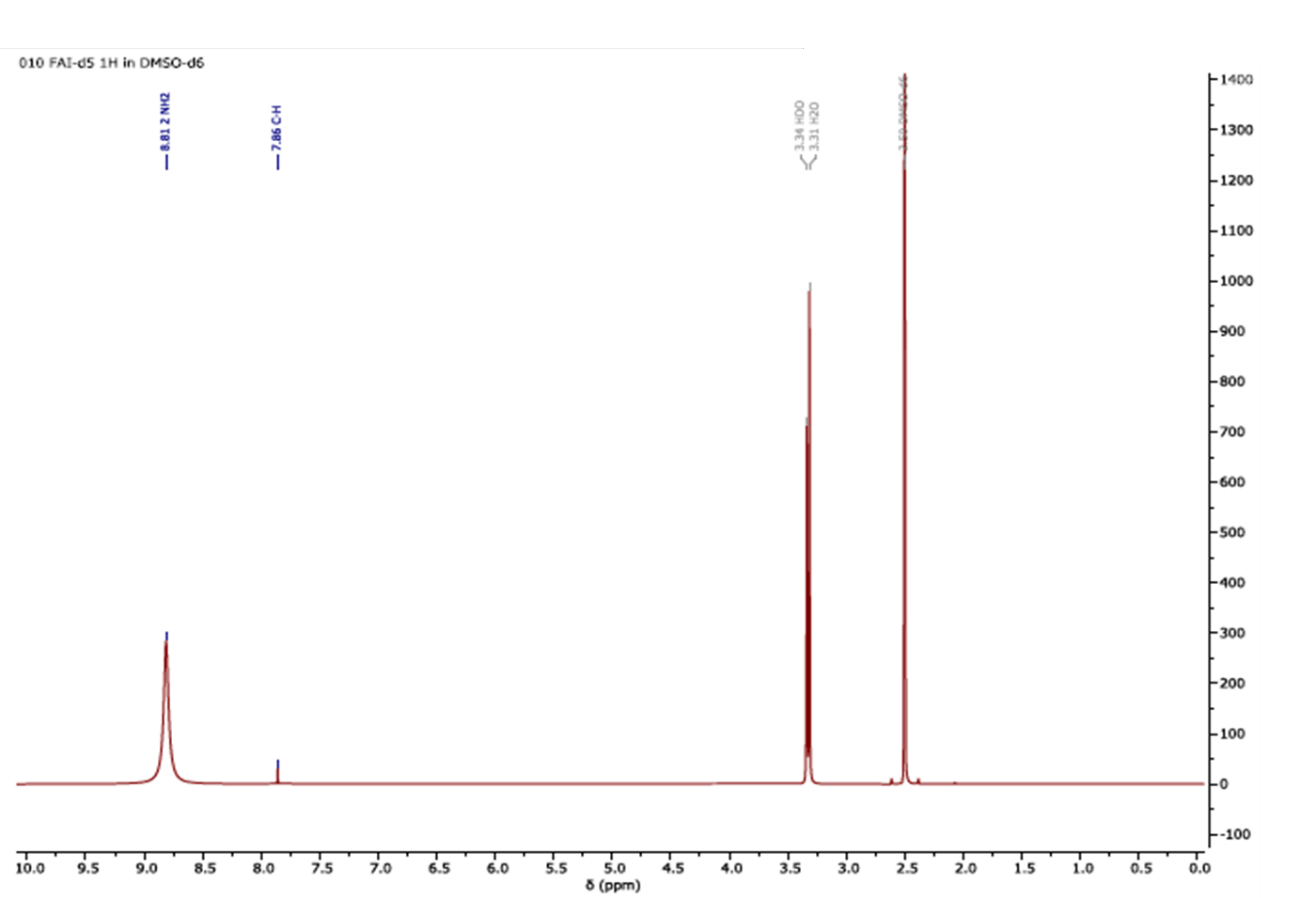
Figure S5.** Static solid-state ^1^H NMR spectrum of FAI-d6 in DMSO-d6 collected at -20 °C.

**
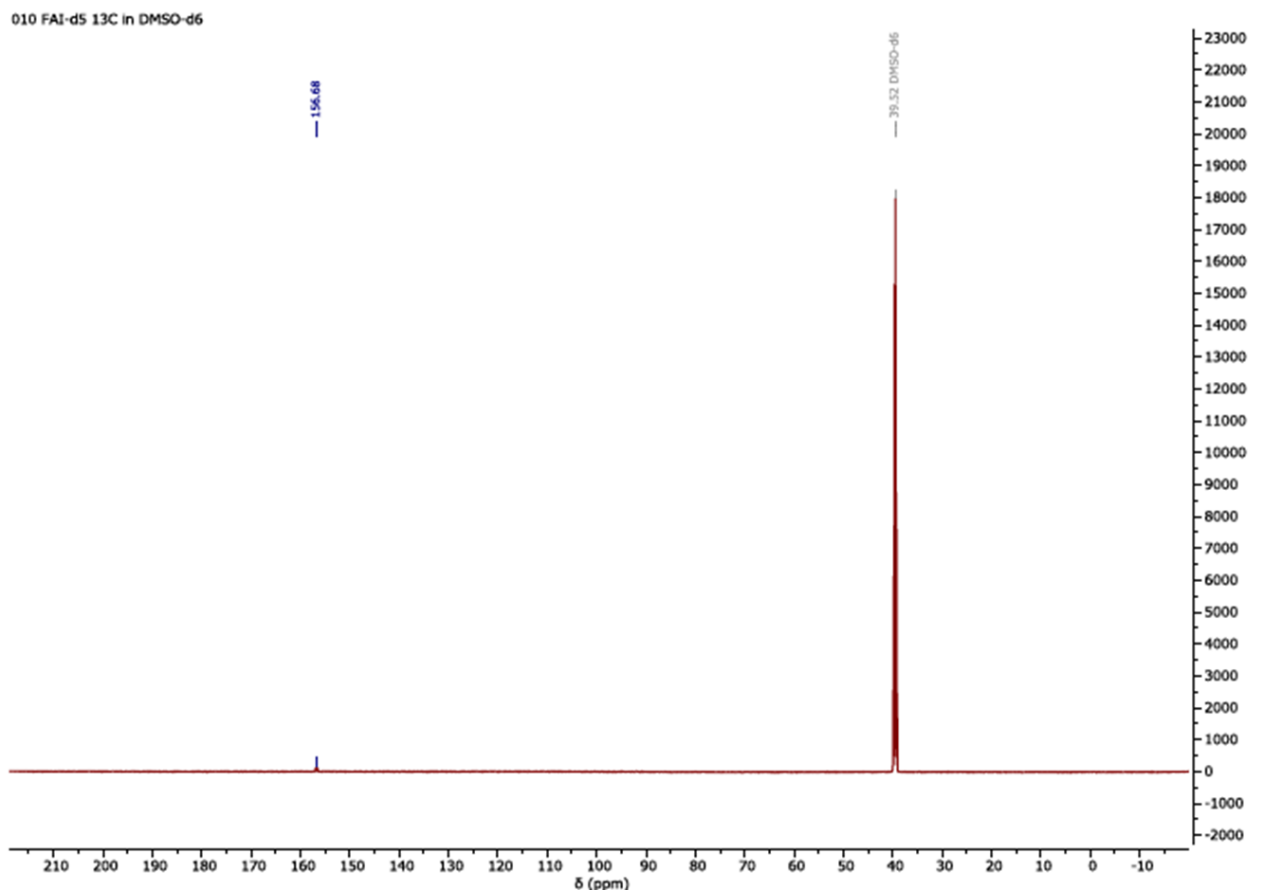
**

**Figure S6.** Static solid-state ^13^C NMR spectrum of FAI-d6 in DMSO-d6 collected at -20 °C.

**
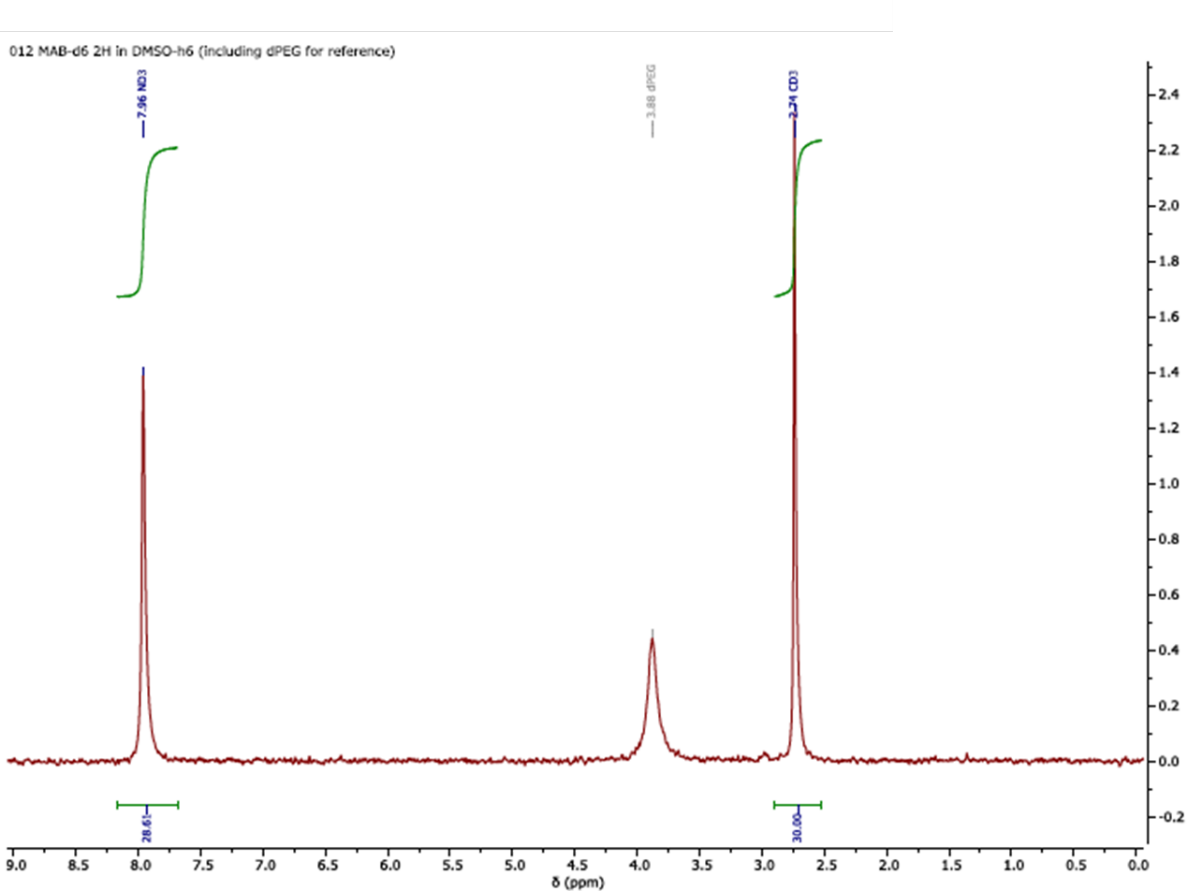
**

Figure S7. Static solid-state ^2^H NMR spectrum of MAB-h6 in DMSO-h6 collected at -20 °C.

**
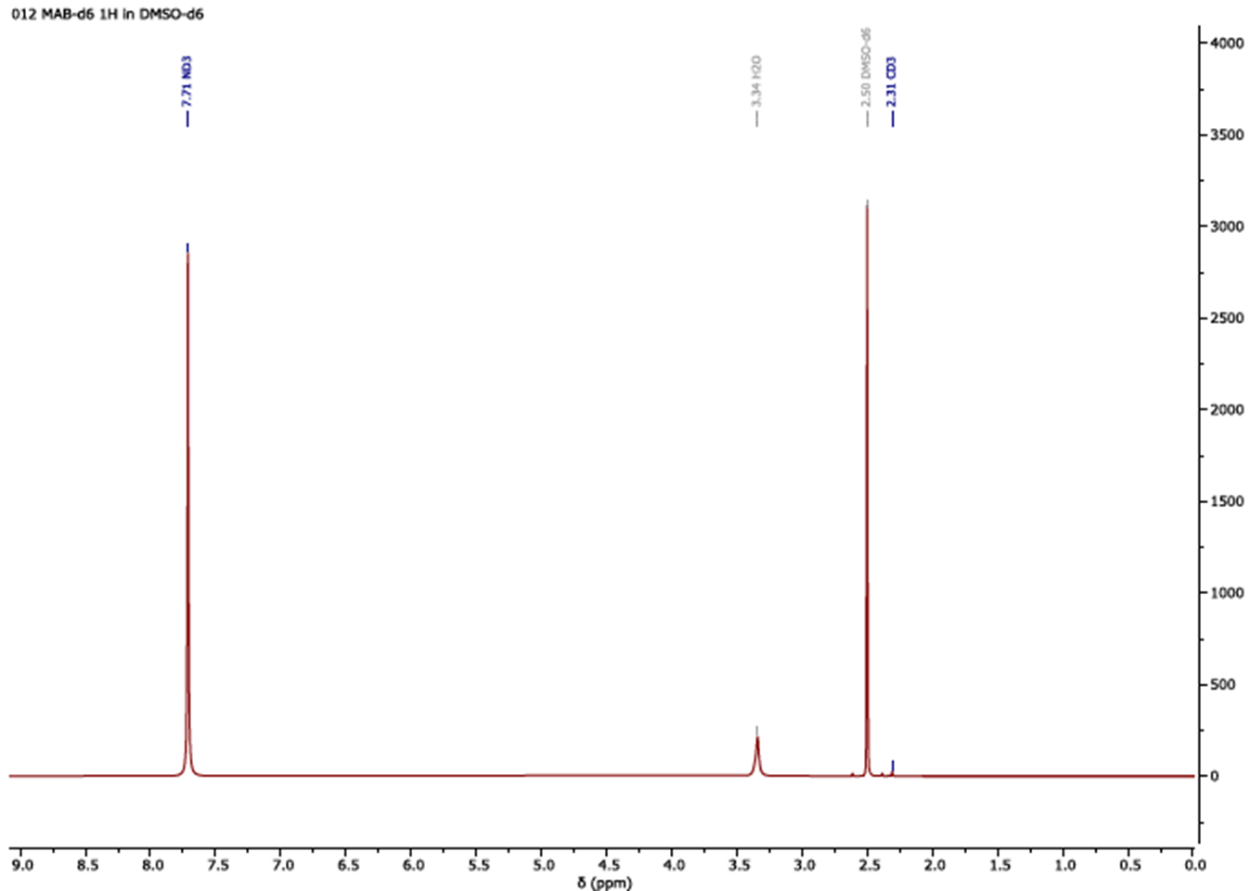
**

**Figure S8.** Static solid-state ^1^H NMR spectrum of MAB-d6 in DMSO-d6 collected at -20 °C.

**
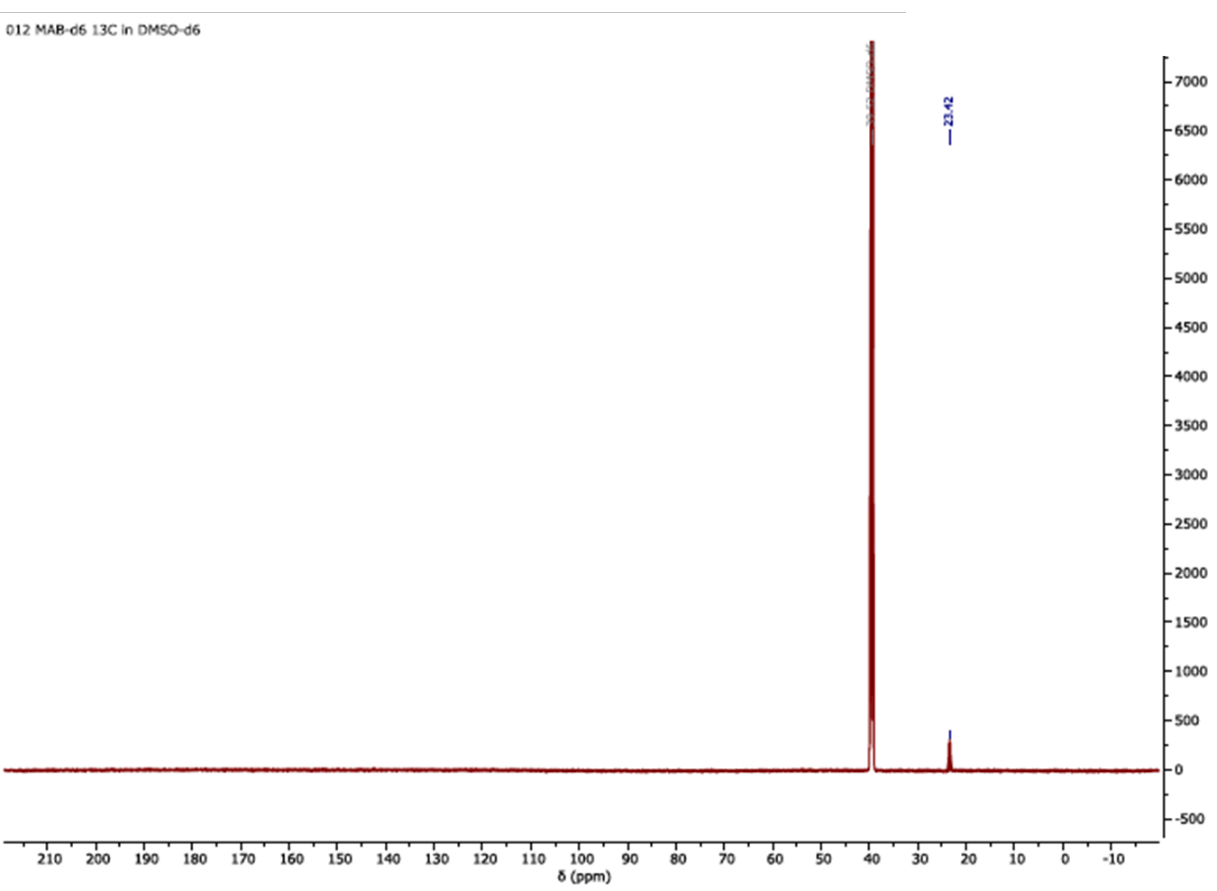
**

**Figure S9.** Static solid-state ^13^C NMR spectrum of MAB-d6 in DMSO-d6 collected at -20 °C.

**Table S1.** Characteristic parameters extracted from the J-V curves. Open circuit voltage (V_OC_) expressed in volts (V), Short circuit current (J_SC_) expressed in (mA·cm^-2^), fill factor (FF) and power conversion efficiency (PCE) expressed in (%).

| Humidity (%RH) | | | | | |
| --- | --- | --- | --- | --- | --- |
|  | **V_OC_** | **J_SC_** | **FF** | **PCE** |  |
| 30 | 1.061±0.019 | 23.82±0.93 | 0.762±0.029 | 19.26±0.24 |  |
| 50 | 1.059±0.018 | 23.82±0.85 | 0.741±0.022 | 18.69±0.22 |  |
| 60 | 1.018±0.015 | 23.39±1.04 | 0.733±0.035 | 17.46±0.38 |  |
| 70 | 1.018±0.014 | 23.24±1.26 | 0.724±0.034 | 17.14±1.12 |  |
| 80 | 0.958±0.031 | 23.12±1.92 | 0.492±0.07 | 10.91±1.18 |  |
| 90 | 0.401±0.074 | 21.84±1.89 | 0.320±0.11 | 2.80±2.14 |  |
| 98 | 0.375±0.104 | 19.27±2.75 | 0.310±0.14 | 2.25±1.67 |  |
| after | 0.294±0.096 | 20.62±2.87 | 0.281±0.04 | 1.71±1.21 |  |

| Temperature (ºC) | | | | |  |
| --- | --- | --- | --- | --- | --- |
|  | **V_OC_** | **J_SC_** | **FF** | **PCE** | |
| 25 | 1.053±0.005 | 23.67±0.28 | 0.76±0.013 | 18.94±0.32 | |
| 30 | 1.063±0.006 | 23.57±0.15 | 0.75±0.024 | 18.77±0.13 | |
| 40 | 1.065±0.003 | 23.27±0.12 | 0.742±0.01 | 18.38±0.27 | |
| 50 | 1.03±0.004 | 23.09±0.92 | 0.729±0.021 | 17.33±0.45 | |
| 60 | 1.02±0.007 | 23.12±0.75 | 0.719±0.02 | 16.95±0.82 | |
| 80 | 0.957±0.018 | 22.00±1.1 | 0.683±0.06 | 13.13±0.13 | |
| 90 | 0.923±0.02 | 20.07±0.9 | 0.625±0.02 | 12.71±0.26 | |
| after | 1.017±0.01 | 22.57±0.87 | 0.676±0.01 | 15.51±0.65 | |


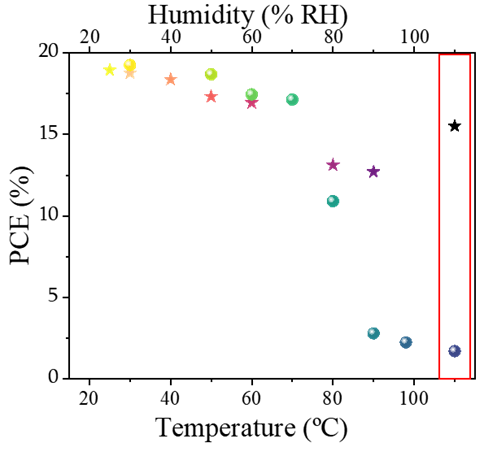


**Figure S10a**. Evolution of the power conversion efficiencies (PCE) extracted from the J-V curves during external thermal and humidity exposure of the prepared solar cell device.


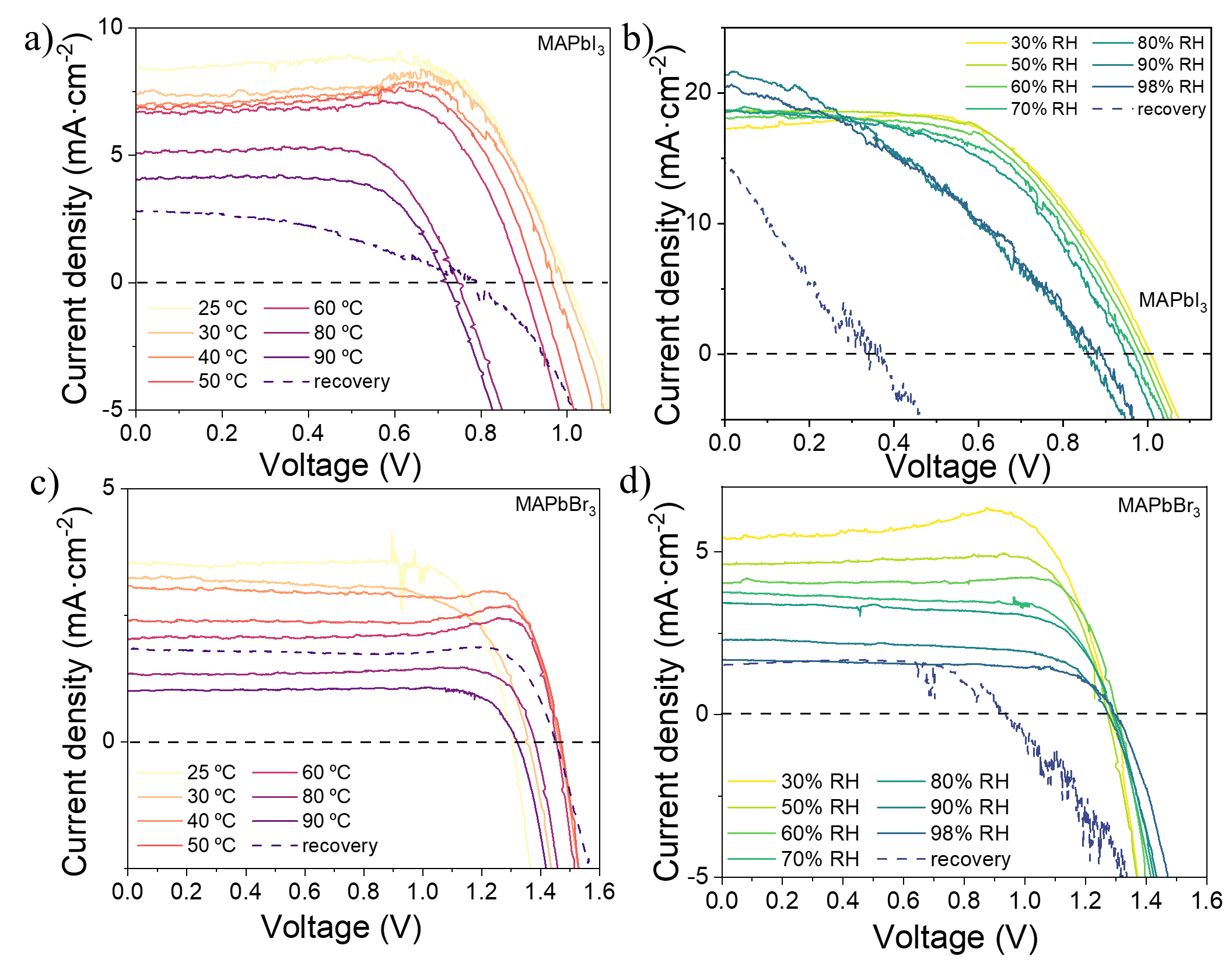


**Figure S10b**. Evolution of the J-V curves during external thermal and humidity exposure (solid lines); and after external humidity and thermal exposure (dotted line) of the prepared solar cell device.


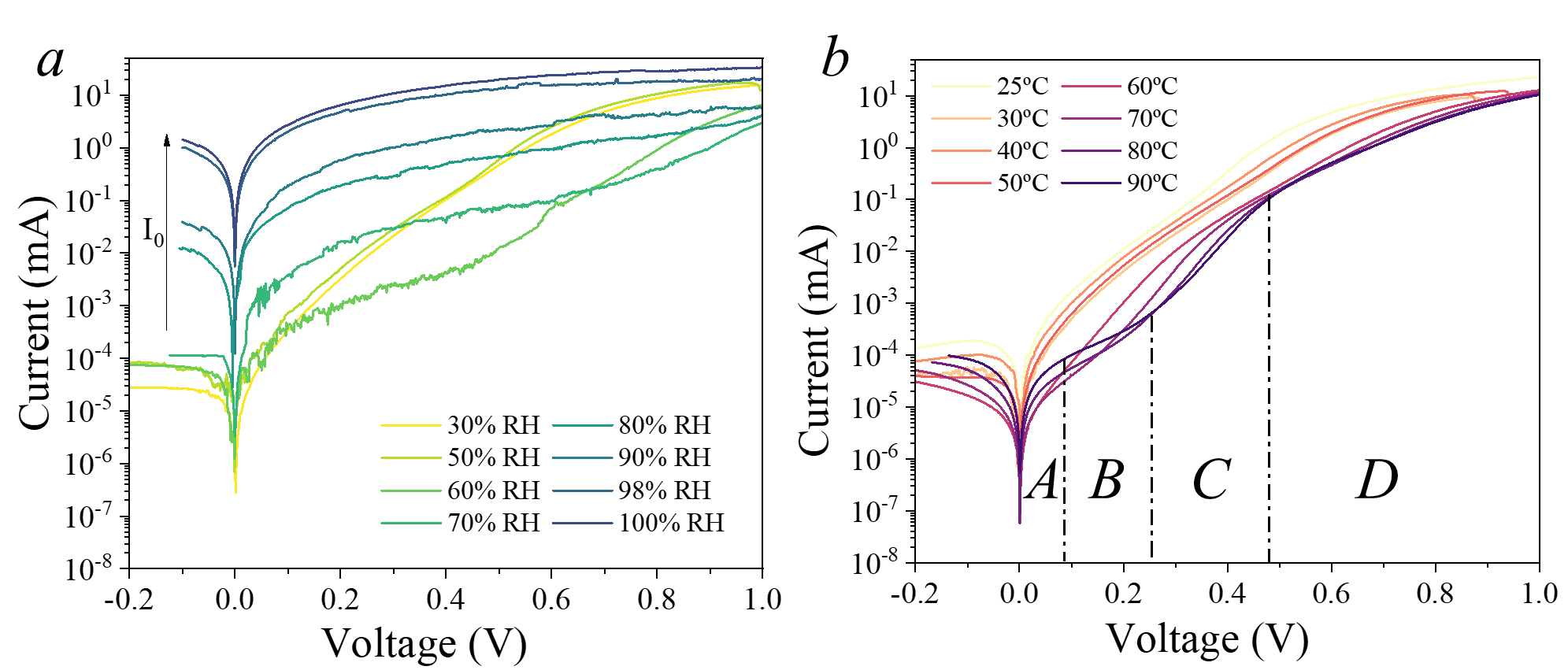


**Figure S11**. a) Dark J-V measurements of perovskite solar cells exposed from 30 to 100 %RH and b) exposed from room temperature to 90 ⁰C.

| **Material** | ***ρ***, 10^-6^ Å^-2^ |
| --- | --- |
| Si | 2.07 |
| SiO_2_ | 3.46 |
| H_2_O | -0.56 |
| PbI_2_ | 1.6 |
| PbBr_2_ | 2.5 |
| TiO_2_ | 2.2-2.4 |
| MAPI | 0.76 |
| FAPI | 1.24 |
| dMAPI | 3.25 |
| dFAPI | 3.23 |
| MAI | -0.10 |
| FAI | 0.84 |
| dMAI | 4.97 |
| *d*FAI | 4.80 |
| MABr | 0.034 |
| dMABr | 5.77 |
| MAPBr | 1.2 |
| dMAPBr | 4.06 |
| (MAPBr)_0.15_(FAPI)_0.85_ | 1.24 |
| (dMAPBr)_0.15_(FAPI)_0.85_ | 1.64 |
| (MAPBr)_0.15_(dFAPI)_0.85_ | 3.9 |

**Table S2.** Calculated neutron scattering length density values *ρ* from the nominal compositions of different components of the studied perovskite films ($\rho=\frac{\left( \sum_{i} N_{i}b_{i} \right)}{V}$)

**Figure S12**. Neutron reflectivity curves for different temperatures measured for dMA_0.15_/FA_0.85_ on Si block at different temperatures. Increasing temperature by ~130K doesn´t result in qualitative changes of perovskite layer structure. For comparison, a more pronounced change is observed under high humidity conditions.


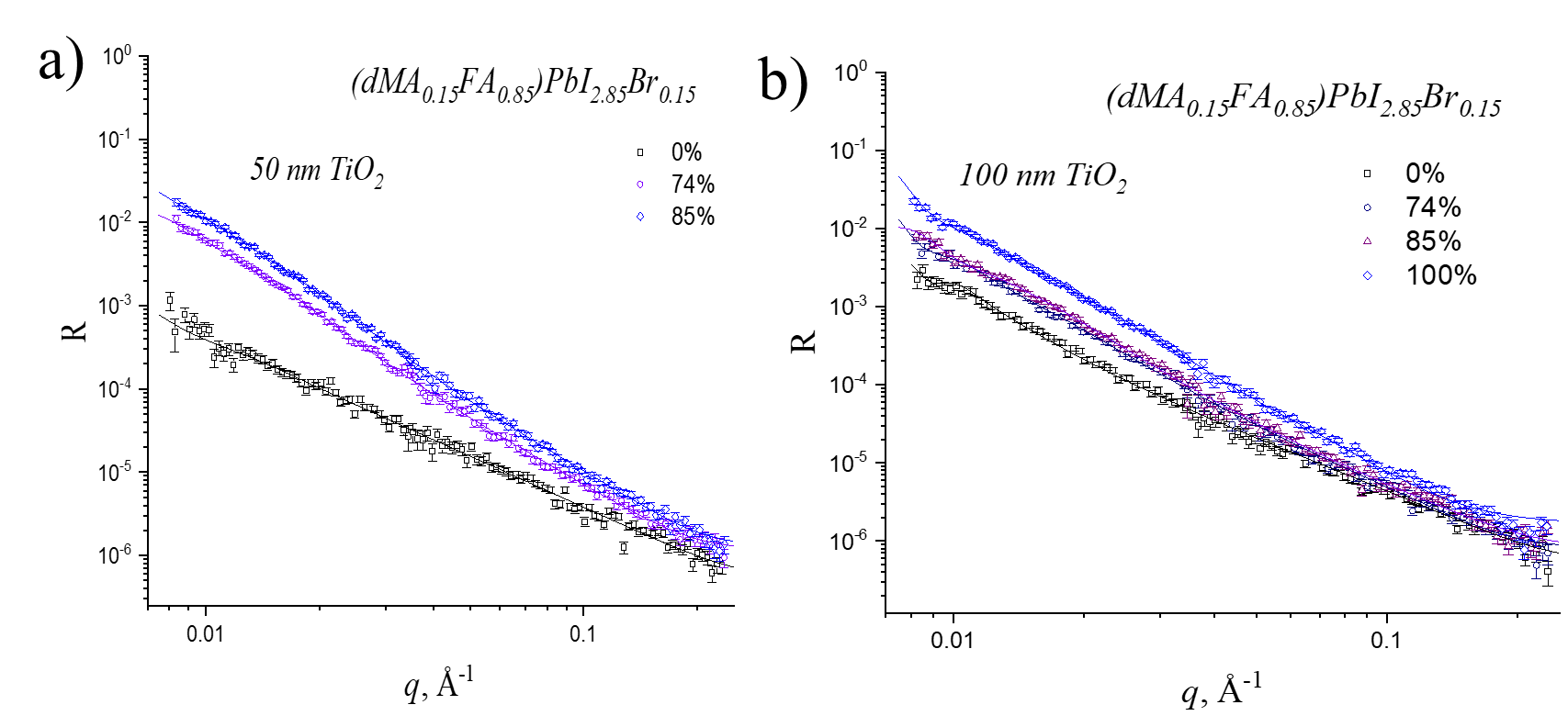


**Figure S13**. Neutron reflectivity curves for different humidities measured for dMA_0.15_/FA_0.85_ on Si/50 nm TiO_2_ and on Si/100 nm TiO_2_.

**Table S3.** Summary of Degradation Rates and Times at Different Humidity Levels

|  |  | FAI | | PbI_2_ | |
| --- | --- | --- | --- | --- | --- |
| ***Humidity (%)*** | ***Temperature (K)*** | ***Rate Constant (k) [molecules/ns]*** | ***Time to Full Degradation (s)*** | ***Rate Constant (k) [molecules/ns]*** | ***Time to Full Degradation (s)*** |
| **20** | 300 | 1.46E-09 | *9.516* | 2.53E-12 | *5088.578* |
|  | 350 | 2.69E-08 | 0.515 | 2.04E-10 | 62.932 |
|  | 400 | 3.23E-07 | 0.043 | 8.61E-09 | 1.495 |
|  | 450 | 2.75E-06 | 5.05E-03 | 2.16E-07 | 5.95E-02 |
|  | 500 | 1.79E-05 | 7.74E-04 | 3.37E-06 | 3.82E-03 |
|  | 550 | 8.86E-05 | 1.57E-04 | 4.76E-05 | 2.70E-04 |
|  | 600 | 3.93E-04 | 3.50E-05 | 3.49E-04 | 3.70E-05 |
| **40** | 300 | 2.92E-09 | 4.758 | 5.06E-12 | 2544.289 |
|  | 350 | 5.39E-08 | 0.257 | 4.09E-10 | 31.466 |
|  | 400 | 6.46E-07 | 0.021 | 1.72E-08 | 0.747 |
|  | 450 | 5.49E-06 | 2.53E-03 | 4.32E-07 | 2.98E-02 |
|  | 500 | 3.58E-05 | 3.87E-04 | 6.74E-06 | 1.91E-03 |
|  | 550 | 1.77E-04 | 7.80E-05 | 9.53E-05 | 1.35E-04 |
|  | 600 | 7.86E-04 | 1.80E-05 | 6.99E-04 | 1.80E-05 |
| **60** | 300 | 4.37E-09 | 3.172 | 7.59E-12 | 1696.193 |
|  | 350 | 8.08E-08 | 0.172 | 6.13E-10 | 20.977 |
|  | 400 | 9.69E-07 | 0.014 | 2.58E-08 | 0.498 |
|  | 450 | 8.24E-06 | 1.68E-03 | 6.48E-07 | 1.98E-02 |
|  | 500 | 5.38E-05 | 2.58E-04 | 1.01E-05 | 1.27E-03 |
|  | 550 | 2.66E-04 | 5.20E-05 | 1.43E-04 | 9.00E-05 |
|  | 600 | 1.18E-03 | 1.20E-05 | 1.05E-03 | 1.20E-05 |
| **80** | 300 | 5.83E-09 | 2.379 | 1.01E-11 | 1272.145 |
|  | 350 | 1.08E-07 | 0.129 | 8.18E-10 | 15.733 |
|  | 400 | 1.29E-06 | 0.011 | 3.44E-08 | 0.374 |
|  | 450 | 1.10E-05 | 1.26E-03 | 8.64E-07 | 1.49E-02 |
|  | 500 | 7.17E-05 | 1.94E-04 | 1.35E-05 | 9.54E-04 |
|  | 550 | 3.54E-04 | 3.90E-05 | 1.91E-04 | 6.80E-05 |
|  | 600 | 1.57E-03 | 9.00E-06 | 1.40E-03 | 9.00E-06 |
| **100** | 300 | 7.29E-09 | 1.903 | 1.26E-11 | 1017.716 |
|  | 350 | 1.35E-07 | 0.103 | 1.02E-09 | 12.586 |
|  | 400 | 1.61E-06 | 8.59E-03 | 4.30E-08 | 2.99E-01 |
|  | 450 | 1.37E-05 | 1.01E-03 | 1.08E-06 | 1.19E-02 |
|  | 500 | 8.96E-05 | 1.55E-04 | 1.69E-05 | 7.63E-04 |
|  | 550 | 4.43E-04 | 3.10E-05 | 2.38E-04 | 5.40E-05 |
|  | 600 | 1.97E-03 | 7.00E-06 | 1.75E-03 | 7.00E-06 |

**Table S4.** Force Field Parameters for the Slab: Charge q (e), interatomic distance σ (nm) and well depth ε (kJ/mol). HGP2 corresponds to the hydrogen atom on the ammonia group of MA (methylammonium), while HGA4 and HGP3 correspond to the hydrogen atoms on the methine group and amine group of FA (formamidinium), respectively.

| ***Charges (q)*** | | |
| --- | --- | --- |
|  | ***Pb*** | 1.3418 |
|  | ***Br*** | -0.6709 |
|  | ***I*** | -0.6709 |
| **MA** | ***N3P3*** | 0.0797 |
|  | ***C334*** | 0.16 |
|  | ***HGA2*** | 0.05 |
|  | ***HGP2*** | 0.08763 |
| **FA** | ***N2P1*** | -0.4405 |
|  | ***C2N1*** | 0.2767 |
|  | ***HGA4*** | 0.15 |
|  | ***HGP3*** | 0.276725 |
|  | ***σ (nm)*** | ***ε (kJ/mol)*** |
| **Br Br** | 0.408 | 1.050184 |
| **Br I** | 0.44 | 1.220474 |
| **Br Pb** | 0.316 | 1.70681 |
| **I I** | 0.472 | 1.418376 |
| **I Pb** | 0.324 | 1.983573 |
| **Pb Pb** | 0.667 | 2.773992 |
| **Br HGP2** | 0.206 | 0.44958 |
| **Br HGP3** | 0.315 | 0.439698 |
| **Br N2P1** | 0.333 | 0.550621 |
| **Br N3P3** | 0.33 | 0.93744 |
| **HGP2 HGP3** | 0.148 | 0.188234 |
| **HGP2 I** | 0.276 | 0.522481 |
| **HGP2 N2P1** | 0.183 | 0.235719 |
| **HGP2 Pb** | 0.385 | 0.73068 |
| **HGP3 I** | 0.276 | 0.510996 |
| **HGP3 N2P1** | 0.291 | 0.230538 |
| **HGP3 N3P3** | 0.293 | 0.392494 |
| **HGP3 Pb** | 0.344 | 0.714619 |
| **I N2P1** | 0.364 | 0.639906 |
| **I N3P3** | 0.35 | 1.089448 |
| **N2P1 N3P3** | 0.491 | 0.491509 |
| **N2P1 Pb** | 0.368 | 0.894897 |
| **N3P3 Pb** | 0.424 | 1.523574 |
